# Population genomics analyses of European ibex species show lower diversity and higher inbreeding in reintroduced populations

**DOI:** 10.1111/eva.12490

**Published:** 2017-10-27

**Authors:** Christine Grossen, Iris Biebach, Samer Angelone‐Alasaad, Lukas F. Keller, Daniel Croll

**Affiliations:** ^1^ Department of Evolutionary Biology and Environmental Studies University of Zürich Zürich Switzerland; ^2^ Laboratory of Evolutionary Genetics Institute of Biology University of Neuchâtel Neuchâtel Switzerland

**Keywords:** bottlenecks, conservation genetics, reintroductions, restriction‐associated DNA sequencing, runs of homozygosity

## Abstract

Restoration of lost species ranges to their native distribution is key for the survival of endangered species. However, reintroductions often fail and long‐term genetic consequences are poorly understood. Alpine ibex (*Capra ibex*) are wild goats that recovered from <100 individuals to ~50,000 within a century by population reintroductions. We analyzed the population genomic consequences of the Alpine ibex reintroduction strategy. We genotyped 101,822 genomewide single nucleotide polymorphism loci in 173 Alpine ibex, the closely related Iberian ibex (*Capra pyrenaica*) and domestic goat (*Capra hircus*). The source population of all Alpine ibex maintained genetic diversity comparable to Iberian ibex, which experienced less severe bottlenecks. All reintroduced Alpine ibex populations had individually and combined lower levels of genetic diversity than the source population. The reintroduction strategy consisted of primary reintroductions from captive breeding and secondary reintroductions from established populations. This stepwise reintroduction strategy left a strong genomic footprint of population differentiation, which increased with subsequent rounds of reintroductions. Furthermore, analyses of genomewide runs of homozygosity showed recent inbreeding primarily in individuals of reintroduced populations. We showed that despite the rapid census recovery, Alpine ibex carry a persistent genomic signature of their reintroduction history. We discuss how genomic monitoring can serve as an early indicator of inbreeding.

## 1 INTRODUCTION

Many species experienced near‐extinction events in their demographic history. Habitat protection and reintroductions are the main strategies for species conservation. The choice of the most suitable reintroduction strategy for a certain species is crucial for the future success and long‐term viability of populations. Despite a growing list of successful species reintroductions that re‐established historic species ranges (Seddon, Armstrong, & Maloney, 2007), the success rate of reintroductions is relatively low. Various aspects such as the ecology (e.g., habitat, diseases) and genetics seem to influence the outcome (Armstrong & Seddon, 2008). Genetic aspects influence not only the short‐term reintroduction success (due to the genetic architecture of the founder individuals), but also the long‐term reintroduction success (due to adaptive evolutionary potential and inbreeding). Several studies identified genetic consequences imposed by reintroductions based on microsatellite loci (e.g., Biebach & Keller, 2009; Diefenbach, Hansen, Bohling, & Miller Butterworth, 2015; Jamieson, 2011; Strzała, Kowalczyk, & Łukaszewicz, 2015), but only very few studies surveyed genomewide effects or monitored long‐term consequences.

Reintroductions are generally composed of two key stages. The first stage is the transfer of individuals and successful reproduction in a new area; such events were causing founder effects in reintroduced populations with a likely loss of genetic diversity. The maintenance of genetic diversity during the first step is a major challenge during species reintroductions (Jamieson & Lacy, 2012; Tracy, Wallis, Efford, & Jamieson, 2011).

The second stage of a reintroduction is the minimization of long‐term demographic effects on populations after the reintroduction event. Major concerns are low effective population size due to slow population growth rates (mainly caused by postrelease mortality and low reproduction rates), lack of gene flow with other populations, and inbreeding. The individuals’ ability to move in space and contribute to gene flow among populations is key for the long‐term survival of reintroduced populations. Hence, reintroductions in a continuous, connected landscape may be less likely to fail than reintroductions in a strongly subdivided landscape due to anthropogenic or topographical factors.

The different stages of a species reintroduction are predicted to show different signatures of genetic subdivisions. At the early stage of species reintroductions, population differentiation is expected to be weak if reintroduced individuals were drawn at random from a source population. Subsequently, restricted gene flow in a fragmented habitat will lead to genetic drift and reduced genetic diversity. The effect of habitat fragmentation is an important factor for the long‐term viability of reintroduced populations. The most immediate consequence of reduced gene flow for a small population is an increase in inbreeding (i.e., mating among related individuals). Inbreeding can have harmful consequences on individual survival and reproductive success (Keller & Waller, 2002). Negative effects of inbreeding on fitness are called inbreeding depression and were widely reported in populations experiencing elevated levels of inbreeding (Keller & Waller, 2002). For instance, in a small island population of song sparrows, several different traits including lifetime reproductive success, survival, and immune response were susceptible to inbreeding (Keller, 1998; Keller, Arcese, Smith, Hochachka, & Stearns, 1994; Reid, Arcese, & Keller, 2003). An increase in inbreeding of 10% led to a decreased male lifetime reproductive success of 25% (Keller, Marr, & Reid, 2006).

Inbreeding depression is an important concern for species conservation (Allendorf, Hohenlohe, & Luikart, 2010; Allendorf, Luikart, & Aitken, 2012; Hoffman et al., 2014; Xue et al., 2015). However, inbreeding depression may only be detectable many generations after an increase in inbreeding. Conservation management can prevent inbreeding depression by translocating individuals from genetically distant populations. Translocations also harbor risks by increasing the likelihood of disease transmission or causing outbreeding depression by breaking up locally adapted allele combinations. Therefore, a careful assessment of the inbreeding risk is an important aspect of successful conservation management actions. Inbreeding is generally estimated using pedigrees (Wright, 1922), which are established from detailed population surveys over multiple generations. In a species with long generation times, pedigree estimations and fitness estimates are difficult to obtain and time‐consuming. Hence, such data are only available for few large mammals (e.g., Overall, Byrne, Pilkington, & Pemberton, 2005; Walling et al., 2011). For conservation management practices, pedigree and fitness estimates are impractical to obtain.

Genetic markers can serve to estimate inbreeding directly without the need for pedigrees. Levels of multilocus heterozygosity can be estimated based on neutral genetic markers (e.g., microsatellites). However, traditional genetic markers such as microsatellites suffer from a number of biases and shortcomings. Microsatellite markers were generally developed to maximize polymorphism in a focal population, which may lead to ascertainment bias in other populations. Recently developed genomic tools based on high‐throughput sequencing such as restriction‐associated DNA sequencing (RAD‐seq) alleviate many of the shortcomings of microsatellite markers. The unbiased genomewide estimates of multilocus heterozygosity generally reflect more accurately levels of inbreeding (Kardos, Taylor, Ellegren, Luikart, & Allendorf, 2016). Recent inbreeding can be estimated by genomewide analyses of runs of homozygosity (ROH, Broman & Weber, 1999; Lencz et al., 2007; Keller, Visscher, & Goddard, 2011; Pemberton et al., 2012). ROH are estimated from physical marker locations along the genome. Long ROH are a strong indicator for recent inbreeding as long ROH indicate that the parental individuals were related. While ROH have been extensively studied in humans and domestic animals (e.g., Lencz et al., 2007; McQuillan et al., 2008; Pemberton et al., 2012; Purfield, Berry, McParland, & Bradley, 2012), this measure has rarely been used to estimate inbreeding as a consequence of reintroductions and species conservation. The recent advances in genome sequencing make it feasible to estimate ROH in many nonmodel species (e.g., Xue et al., 2015). Estimating inbreeding based on ROH rather than pedigrees has several advantages (Kardos, Luikart, & Allendorf, 2015; Keller et al., 2011). ROH estimate the fraction of the genome that is identical by descent (i.e., autozygous) and serve to estimate the age of inbreeding events. The power to detect early signs of inbreeding through ROH may help to prevent inbreeding depression before reaching detectable fitness consequences in populations.

Alpine ibex (*Capra ibex*) are an ideal system to test for the long‐term population genetic consequences of population reintroductions. Alpine ibex are a species of wild goat living at high elevation across the European Alps. The species was overhunted over centuries and went nearly extinct in the early 19th century. An extensive conservation program started in 1906 that reintroduced Alpine ibex to their original distribution range re‐establishing the census size from ca. 100 individuals to the current census of ca. 50,000 (Brambilla pers. comm.). The reintroduction of Alpine ibex to the European Alps was likely the most successful reintroduction of a large mammal. After recovery of the sole remaining population in the Gran Paradiso area of northern Italy to well over 1,000 individuals, 55 male and 45 female ibex were transferred from the Gran Paradiso area to two Swiss zoos to initiate a captive breeding program (Giacometti, 2006). At least 11 individuals died before reproducing, and thus, the maximum captive founder stock consisted of 88 individuals (Stuwe & Nievergelt, 1991). The individuals were first bred in captivity in two Swiss zoos. As all individuals used for the captive breeding originated from the same source population (Gran Paradiso), the zoo‐bred population likely started with no genetic subdivisions. Offspring of the zoo‐bred population were introduced to different locations in the Swiss Alps. Three reintroduced populations reproduced well (here called “primary” populations) and served as source populations for multiple, secondary reintroductions (here called “secondary” populations). Most of these secondary reintroductions were each initiated with individuals from a single primary population. Secondary populations originating from more than one source population are here called mixed populations (see Table S1 for more details on the reintroductions). Microsatellite‐based analyses showed that the stepwise reintroduction strategy left a strong genetic footprint in extant populations (Biebach & Keller, 2009). The repeated establishment of new Alpine ibex populations from previously established populations caused up to four serial bottlenecks (captive breeding, primary, one or more secondary reintroductions). Bottlenecks increase the likelihood of inbreeding due to the reduction in effective population size and increase the risk of inbreeding depression. Indeed, populations with higher levels of inbreeding grew more slowly in harsh environments (Bozzuto pers. comm.). In the Gran Paradiso population, heterozygosity and fitness‐related traits were negatively correlated, which suggests that inbreeding can indeed lower fitness (Brambilla, Biebach, Bassano, Bogliani, & von Hardenberg, 2015). Despite effective management and hunting restrictions across the European Alps, some Alpine ibex populations recently declined in size.

The precisely documented reintroduction strategy and the large number of available samples provide a framework to test multiple hypotheses on the genetic trajectory of reintroduced populations. The Gran Paradiso source population can still be sampled, and hence, genetic diversity in reintroduced populations can be compared to a population that was maintained over centuries. We will use the term autochthonous for such extant, nonreintroduced populations. The primary and secondary population reintroduction history is well documented. Hence, changes in genetic diversity and inbreeding can be assessed as a function of the reintroduction history. Furthermore, the population genetic diversity and structure of a related European ibex species can be used as a comparison. The Iberian ibex (*Capra pyrenaica*) is the closest relative to Alpine ibex (Pidancier, Jordan, Luikart, & Taberlet, 2006) and was historically found throughout the Iberian Peninsula, from Portugal to southwest France (Grubb, 2005). Similar to Alpine ibex, Iberian ibex suffered from population bottlenecks due to habitat fragmentation and overhunting over the last two centuries (Perez et al., 2002). About ten small populations survived and were legally protected from 1900 to 1960. From 1960s onwards, several Iberian ibex populations were founded by reintroductions (Perez et al., 2002; Refoyo, Olmedo, & Muñoz, 2016). Present‐day Iberian ibex populations are mainly found in the mountain ranges of the Sierra Nevada and the Iberian System. Two consecutive and severe bottlenecks (ca. 30 individuals) were reported for the Maestrazgo population between 1940 and 1960 (Couturier, 1962). The Maestrazgo population recovered to 7,000 individuals (Perez et al., 2002). The Sierra Nevada population suffered from a population bottleneck of ca. 600 individuals in the 1960s and is currently the largest Iberian ibex population with about 16,000 individuals (Couturier, 1962; Perez et al., 2002). Sierra Nevada is also thought to be the genetically most diverse population (Manceau, Crampe, Boursot, & Taberlet, 1999). Despite severe demographic changes over the past century, Iberian ibex were not affected by similar range and census reductions as Alpine ibex. The less severe bottlenecks experienced by Iberian ibex are reflected in higher genetic diversity at the major histocompatibility complex (MHC) (Alasaad et al., 2012; Amills et al., 2004).

We aimed to identify the population genomic consequences of the Alpine ibex reintroduction strategy using Iberian ibex and domestic goat (*Capra hircus*) as a reference. We addressed the following questions: (i) What was the loss of genomewide diversity due to the reintroduction of Alpine ibex from a single source population? How do the levels of genetic diversity compare with the major Iberian ibex populations? (ii) Did the stepwise reintroduction strategy of primary and secondary populations lead to a stepwise increase in genetic subdivision with subsequent rounds of reintroductions? (iii) Did the reintroductions lead to genomewide signatures of recent inbreeding? Finally, we discuss how genomewide data can be used to assess the long‐term viability of reintroduced species.

## 2 MATERIALS AND METHODS

### 2.1 Sampling

We obtained 190 samples from 10 Alpine ibex populations, two Iberian ibex (*C. pyrenaica hispanica*) populations, and four Swiss domestic goat breeds. Nine Alpine ibex populations were reintroduced to the Swiss Alps over the past century, and one was the population from the Gran Paradiso National Park in Italy. The sample size per population was 15 for the reintroduced Alpine ibex and Iberian ibex populations, 16 for the source population Gran Paradiso, and two to three per domestic goat breed (Tables 1 and S2).

**Table 1:** Genetic diversity of domestic goat breeds, Alpine ibex, and Iberian Ibex populations. The sequencing coverage is shown as the median base pairs sequenced at restriction sites. The SNP density is shown as SNPs per kilobase sequenced. The estimated SNP density based on resampling corresponds to *n *=* *10 individuals per population

Species	Population/breed	*N*	Base pairs sequenced (median)	Number of SNPs	SNP density (kb^−1^)	SNP density resampling (kb^−1^)	Expected heterozygosity	Observed heterozygosity	Inbreeding coefficient (*F*_IS_)
Alpine ibex	Gran Paradiso (autoch)	12	7,397,057	13,341	1.8	1.72	0.032	0.031	0.020
	Albris (primary)	15	8,357,278	11,369	1.4	1.25	0.024	0.023	0.020
	Brienzer Rothorn (primary)	14	7,257,683	10,573	1.5	1.28	0.024	0.024	0.015
	Pleureur (primary)	15	8,184,974	11,398	1.4	1.22	0.025	0.024	0.025
	Aletsch Bietschhorn (secondary mixed)	13	8,963,724	11,278	1.3	1.17	0.026	0.026	−0.005
	Schwarz Mönch (secondary mixed)	15	9,127,840	10,925	1.2	1.12	0.024	0.022	0.086
	Rheinwaldhorn (secondary)	12	8,010,178	10,166	1.3	1.18	0.023	0.019	0.153
	Graue Hörner (secondary)	10	8,354,074	9,602	1.1	1.15	0.024	0.021	0.116
	Cape au Moine (secondary)	15	10,997,552	11,771	1.1	0.99	0.023	0.025	−0.065
	Weisshorn (secondary)	14	10,720,495	11,518	1.1	1.01	0.024	0.025	−0.057
Iberian ibex	Sierra Nevada (autoch)	15	7,226,088	13,351	1.8	1.55	0.039	0.041	−0.058
	Maestrazgo (autoch)	15	10,122,682	17,766	1.8	1.41	0.040	0.040	0.001
Domestic goat	ALP	3	7,368,844	22,376	3.0		0.196^a^	0.195^a^	0.006^a^
	GRS	2	6,862,879	20,759	3.0				
	PCG	1	5,132,452	7,654	1.5				
	SGB	2	8,904,005	27,899	3.1				

a Due to the low sample size per breed, these statistics were calculated for the pool of all breeds.

### 2.2 RAD library preparation and SNP calling

Genomic DNA was extracted from tissue or blood samples using a BioSprint 96 kit (QIAGEN). The RAD libraries were prepared as described in Grossen, Keller, Biebach, and Croll (2014). In short, 1.35–1.5 μg of DNA was digested using the restriction enzyme *Sbf*I (New England Biolabs). Adapters containing unique barcodes were ligated to the digested DNA. Ligated DNA was sheared using a COVARIS ultrasonicator and size‐selected at 300–700 bp on a CALIPER LabChip XT. After ligation of P2 adapters, a PCR was performed to amplify the library. Quality‐checked libraries were sequenced on an Illumina HiSeq 2000 platform to generate 100‐bp paired‐end reads. We obtained a total of 468 million high‐quality reads and a median of 2.3, 2.1, and 1.6 mio read pairs per Alpine ibex, Iberian ibex, and domestic goat individual, respectively (Fig. S1).

Raw sequencing reads were demultiplexed using the FASTX‐toolkit ( http://hannonlab.cshl.edu/fastx_toolkit/index.html) according to the P1 adapter barcode. Trimmomatic (Bolger, Lohse, & Usadel, 2014) was used for quality and Illumina adapter trimming, as well as the removal of the RAD‐seq barcodes. Reads were aligned to the reference genome of the domestic goat using Bowtie2 version 2.2.4 with “end‐to‐end” and “very‐sensitive” settings (Langmead & Salzberg, 2012). The domestic goat reference genome was trimmed for contigs <1 kb (ref_CHIR_1.0, Dong et al., 2012; downloaded from ftp.ncbi.nih.gov). Read alignments were used for multisample SNP genotyping as follows. Genotype calling was performed using HaplotypeCaller and GenotypeGVCFs according to best practices (GATK, version 3.3.0; DePristo et al., 2011; McKenna et al., 2010). SNPs were hard‐filtered using VariantFiltration and SelectVariants. SNPs were removed if any of the following criteria were met: QD < 2.0, MQ < 30.0, −12.5 > MQRankSum > 12.5, FS > 60.0, ReadPosRankSum < −8.000, QUAL < 30.0, AN < 40. Vcftools (Danecek et al., 2011) was used for all further filtering steps. Genotypes with genotype quality (GQ) <20 were set to missing. A total of 16 individuals were removed from the dataset due to low read number (less than 900,000) and/or high missing rates (higher than 80% for unfiltered SNPs). One additional individual was removed due to very high levels of heterozygosity (0.24), which could neither be explained by introgression nor gene flow among divergent populations (see Table S2). Autosomal SNPs were retained if the genotyping rate was at least 45%. Furthermore, we filtered out potential paralogous regions by requiring that the normalized coverage was less than twofold the mean coverage of other SNPs (normalized by individual). The resulting SNP panel was called “general.” Further filters were applied to produce different SNP panels aimed at maximizing the information content for different phylogenetic and population genetic analyses. For phylogenetic and comparative population genomic analyses among species, the SNP panel “multispecies” was produced. For this, only SNPs with a genotyping rate of at least 50% within each species and 60% across species were retained. The SNP panel “intraspecies” was produced for all analysis performed for each species individually. For this, SNPs with a genotyping rate of >80% within a given species were retained. For all panels, monomorphic SNPs within a given group were removed (i.e., among all species together for “multispecies” or per species for “intraspecies”), as well as if the minor allele was found in only one individual (singletons and private doubletons).

### 2.3 Phylogenetic and genetic diversity analyses among species

Two maximum‐likelihood phylogenetic trees were built using RAXML (7.3.0, Stamatakis, 2006) based on a supermatrix of the “multispecies” SNP panel. The first tree explored the phylogenetic relationships among the three species and included one Alpine ibex, one Iberian ibex of each of the two populations, and one domestic goat. Two individuals representing Iberian ibex were used for this first tree because a relatively high differentiation was expected between the two populations from a previous study (Angelone‐Alasaad pers. comm.). A second tree explored the evolutionary relationship among all Alpine and Iberian ibex in our sample. We applied the GTR+gamma model for sequence evolution. A rapid bootstrap analysis including the search of the best maximum‐likelihood tree was run with 100 replicates (Stamatakis, Hoover, & Rougemont, 2008). The trees were visualized using FigTree v1.4.2 ( http://tree.bio.ed.ac.uk).

The R package {SNPRelate} was used to perform a principal component analysis (PCA) of all three species based on the genetic covariance matrix calculated from the genotypes (Zheng et al., 2012). For this, we used the SNP panel “multispecies” but retained only individuals genotyped at least at 80% of all SNPs (94 individuals). SNPs were then further filtered for a genotyping rate >80% and a minor allele frequency (MAF) of >5%. A total of 10,881 SNPs were retained. These additional filters were applied to avoid potential confounding effects of variable genotyping rates (see also Fig. S2C–F).

### 2.4 Population structure within species

Principal component analysis as described above were also performed for each species individually. For this, the SNP panels “intraspecies” were used. As above, only individuals genotyped at least at 80% of all SNPs were retained (100 individuals) and SNPs were further filtered for a genotyping rate >80% and a minor allele frequency (MAF) of >5%. The function stat_ellipse() in the R package {ggplot2} was used to draw a 95% confidence ellipse for each population assuming a multivariate *t*‐distribution.

For a complementary analysis of population structure, we used the Bayesian clustering method STRUCTURE (Pritchard, Stephens, & Donnelly, 2000). The same marker and individual set was used as for the PCA except for requiring a minimum of 250 kb pairwise distance between adjacent SNPs (3,167 SNPs left) to reduce potential nonindependence among loci. We varied *K* from *K* = 2 to *K* = 4 and ran for each *K* a burn‐in of 10,000 and 50,000 Monte Carlo replicates using an admixture model with correlated allele frequencies.

For a quantitative assessment of the population structure of Alpine ibex, we performed a hierarchical AMOVA as implemented in the R package {poppr} with the method ade4. With the exception of two, all secondary reintroduced populations in this study were established from one primary reintroduced population. For the lower hierarchical level, we grouped each secondary reintroduced population with its primary population if there was such a simple 1‐to‐1 relationship. For the higher hierarchical level, we grouped all 1‐to‐1 pairs of reintroduced populations mentioned above and compared these with the source population (Gran Paradiso).

### 2.5 Genetic diversity

The SNP density per population and per species, respectively, was calculated as the number of SNPs per kilobase sequenced with the SNP panel “general.” As variation in sequencing coverage among populations could lead to variation in the number of SNPs passing the filters described above, we decided to use SNP density instead of SNP number to control for variation in sequence coverage. RAD‐seq most consistently covers the region around the defined restriction enzyme cut sites. Hence, we restricted our genetic diversity analyses to these regions. Cut sites were identified in the goat genome by an in silico digest using *restrict* from EMBOSS (Rice, Longden, & Bleasby, 2000). Bedtools (bedtools.readthedocs.org) was used to calculate coverage at the *Sbf*I cut sites. We retained SNPs (excluding singletons and private doubletons) within 90 bp from a cut site. To calculate the SNP density (SNP/kb), the number of SNPs identified in each population (minor allele count ≥1) was divided by the median sequence length covered by at least seven reads in the population. As a cut site is always sequenced in both directions, the effective coverage at the restriction site was 14.

As sample sizes differed among species and populations, we performed a resampling of the SNP density of randomly selected individuals. The different groups for which we performed a resampling were as follows: domestic goat, Iberian ibex from Maestrazgo, Iberian ibex from Sierra Nevada, reintroduced Alpine ibex, and Alpine ibex only from the autochthonous Gran Paradiso population. For each group, we randomly sampled 100 times without replacement subgroups of 1 to *n *− 1 individuals with *n* being the sample size per group. For each random sample, we counted the number of SNPs with a minor allele count of ≥1. Then, the SNP count was divided by the median kilobase sequenced at a coverage of at least 7.

The expected multilocus heterozygosity per populations (or Nei's genetic diversity; Nei, 1973) and the observed multilocus heterozygosity per individual were estimated using vcftools. For this, we used the SNP panel “multispecies” except for only retaining SNPs genotyped in 90% of all individuals. The additional filtering was performed to avoid potential confounding factors due to variable missingness rates among populations (see also Fig. S3).

### 2.6 Estimates of recent individual inbreeding

Runs of homozygosity (ROH) are defined as tracts of homozygosity along physical positions of a chromosome. ROH arise if both parents share identical chromosomal segments (i.e., segments of identity by descent, Lencz et al., 2007; Pemberton et al., 2012). Long ROH can be used to estimate the inbreeding history of individuals in a population (e.g., McQuillan et al., 2008). To identify ROH, we used the SNP panel “multispecies” but retained only individuals genotyped at more than 80% of the SNPs. Furthermore, only SNPs genotyped in ≥90% of the individuals were retained. The remaining dataset contained 41,907 SNPs and 94 individuals. This filtering reduced confounding effects of variable rates of missingness among populations (Fig. S4).

We identified chromosomal tracts composed of ROH using the option –homozyg in Plink (Purcell et al., 2007) as follows: We used a sliding window of 25 SNPs (homozyg‐window‐snp 25). The window was defined as homozygous if there was not more than one heterozygous site (homozyg‐window‐het 1) and not more than five missing sites (homozyg‐window‐missing 5). If at least 5% of all windows that included a given SNP were defined as homozygous, the SNP was defined as being in a homozygous segment of a chromosome (homozyg‐window‐threshold .05). This threshold was chosen to ensure that the edges of a ROH are properly delimited. A homozygous segment was defined as a ROH if all of the following conditions were met: the segment included ≥25 SNPs (homozyg‐snp 25), covered ≥1,000 kb (homoz‐kb 1,000), and contained a maximum of one heterozygous site (homoz‐het 1). Furthermore, the minimum SNP density was one SNP per 50 kb (homozyg‐density 50) and the maximum distance between two neighboring SNPs was ≤1,000 kb (homozyg‐gap 1,000).

### 2.7 Effective population size in recent past

We estimated effective population size using the LD‐based method (Waples & Do, 2008) implemented in NeEstimator V2.01 (Do et al., 2014). SNP panel “general” was used with an additional missingness filter of ≤10% for a given population. The intermarker distance had to be at least 250 kb to avoid strong nonindependence of markers.

## 3 RESULTS

**Figure 1:**
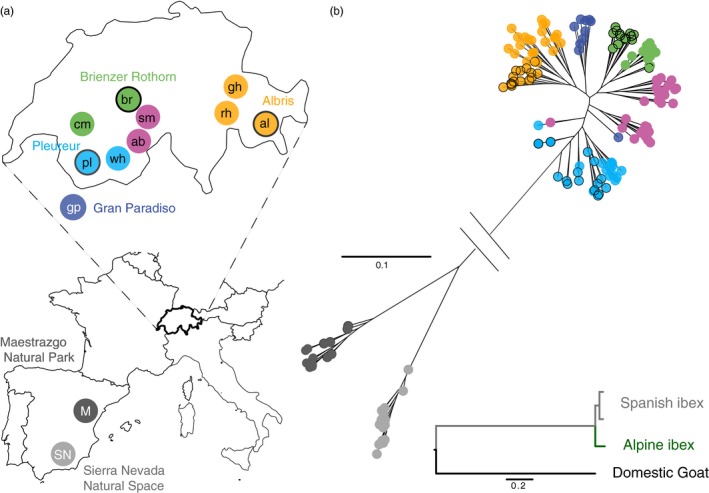
Sampling locations and phylogenetic relationship of Iberian and Alpine ibex. (a) The maps show sampling locations in Spain (lower map) and Switzerland (upper map). Each filled circle represents one population. Black circles around colors indicate the three primary reintroduced populations Albris (orange), Pleureur (light blue), and Brienzer Rothorn (green). Secondary reintroductions established from the primary reintroduced populations share the same color. Populations with mixed ancestry are shown in purple. The autochthonous populations are Gran Paradiso (dark blue), Maestrazgo (dark gray), and Sierra Nevada (light gray). Ibex populations: gp, Gran Paradiso; al, Albris; br, Brienzer Rothorn; pl, Pleureur; ab, Aletsch Bietschhorn; sm, Schwarz Mönch; cm, Cape au Moine; gh, Graue Hörner; rh, Rheinwald; wh, Weisshorn; SN, Sierra Nevada; M, Maestrazgo. (b) Maximum‐likelihood phylogeny based on a supermatrix of SNPs generated by restriction‐associated DNA sequencing of Alpine ibex and Iberian ibex (upper tree) and species relationship among Alpine ibex, Iberian ibex, and domestic goat (lower tree) based on one individual per species (one individual for each Iberian ibex population)

We used RAD‐sequencing to generate high‐density, genomewide markers for Alpine ibex from the Gran Paradiso source population, the three primary Alpine ibex populations established during the reintroductions (Albris, Brienzer Rothorn, Pleureur) as well as subsequently established secondary populations (Figure 1a, Tables S1 and S2). We included Iberian ibex individuals of the subspecies *C. pyrenaica hispanica* from two populations: Maestrazgo Natural Park (north‐eastern Spain) and Sierra Nevada Natural Space (southern Spain, Figure 1a). Both populations were affected by bottlenecks but no extinction events (“autochthonous” populations). We also analyzed domestic goat from four Swiss breeds.

Using RAD‐seq, we obtained overall read alignment rates varying from 95% to 96% among species. The more stringent alignment rates (read pairs aligned concordantly exactly one time) varied between 82% (domestic goat), 84% (Iberian ibex) and 85% (Alpine ibex). As the alignment rates obtained from samples of the different species were highly similar, we expected no or only minimal ascertainment bias in genotype calling among species. All mapped reads were used for SNP calling, and we obtained a total of 101,822 autosomal SNPs (at cut sites), which had the rare allele shared among at least two individuals and an overall genotyping rate of >45%. Our paired‐end RAD‐seq approach enabled the identification of optical and PCR duplicates. The proportion of reads detected as optical duplicates ranged from 0.228 to 0.583 among individuals and the proportion of reads detected as PCR duplicates ranged from 0.232 to 0.312. To test whether the removal of all duplicates (both optical and PCR duplicates) had an impact on population differentiation parameters, we performed an additional principal component analysis. The differentiation of ibex populations was not discernably different if only reads devoid of duplicates were used (see Fig. S5). The mean genotyping rate (at cut sites) per individual was 69% for Alpine ibex (*SD* = 13%), 68% for Iberian ibex (*SD* = 13%), and 61% for domestic goat (*SD* = 9%). The number of SNPs (at cut sites) polymorphic within species was 20,744 in Alpine ibex (*n *=* *135), 24,018 in Iberian ibex (*n *=* *30), and 46,300 in domestic goat (*n *=* *8). The median base pairs covered at restriction sites was 8.9, 8.3, and 7.6 Mb for Alpine ibex, Iberian ibex, and domestic goat, respectively. The coverage amounted to 0.28%–0.33% of the total genome. The median SNP density was 2.3, 2.9, and 6.0 SNPs per kilobase sequenced for Alpine ibex, Iberian ibex, and domestic goat, respectively.

### 3.1 Phylogenetic relationships and genetic differentiation among European ibex

As expected, maximum‐likelihood phylogenetic analyses showed that Alpine and Iberian ibex were closely related (Figure 1b, lower panel). Genotypes of Iberian ibex were strongly differentiated and clustered according to their population of origin (Figure 1b, upper panel). In contrast, Alpine ibex genotypes had overall shorter branch lengths indicating higher relatedness among populations (Figure 1b, upper panel). As in Iberian ibex, Alpine ibex individuals mostly clustered according to their population of origin. Furthermore, primary populations (Brienzer Rothorn, Pleureur, Albris) clustered closely with their respective secondary populations (indicated by colors; Figure 1b).

A principal component analysis clearly separated all three species (Fig. S2A). The first principal component (PC) explained 59.7% of the variation and differentiated all species. The second PC (6.8%) separated domestic goat from the two ibex species. Among ibex, the PCA clearly differentiated the two Iberian ibex populations. Alpine ibex were grouped in a single tight cluster suggesting low within‐species genetic diversity.

**Figure 2:**
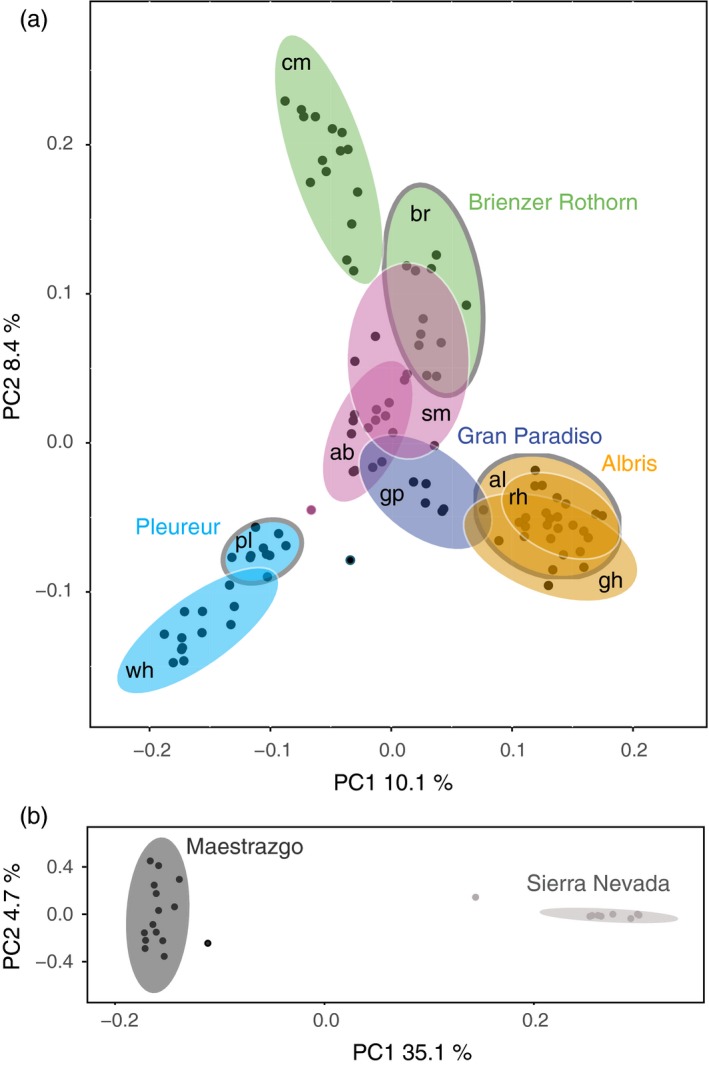
Principal component analysis (PCA) of within‐species diversity. (a) PCA of within‐species and among‐population structure of Alpine ibex. The 95% confidence ellipse per population is shown. Gran Paradiso is the source population of all extant populations. Dark circles show the three primary reintroduced populations Albris (orange), Pleureur (light blue), and Brienzer Rothorn (green). Secondary reintroductions established from the primary reintroduced populations share the same color. Populations with mixed ancestry are shown in purple. Populations abbreviated as in Figure 1a. (b) PCA showing the population structure of Iberian ibex. The 95% confidence ellipse is shown for each population

A PC analysis, performed for Iberian ibex alone, identified two distinct groups without any overlap (Figure 2b, pairwise *F*_ST_ = 0.39). PC1 (35.1%) clearly separated the two populations and therefore explained considerably more variation than PC2 (4.7%). Individuals from the Sierra Nevada population were mostly differentiated by PC1, while individuals from the Maestrazgo population were differentiated by both PC1 and PC2. A PC analysis for domestic goats alone showed a clustering according to breeds (Fig. S2B).

### 3.2 Genetic structure of reintroduced Alpine ibex populations

The extant differentiation among reintroduced Alpine ibex populations should reflect both the genetic diversity of founder individuals and demographic effects experienced by the populations since the reintroduction. Hence, we analyzed the population structure of the Gran Paradiso source population, and the primary and secondary reintroduced populations. The PCA of Alpine ibex populations showed a pronounced substructure with three differentiated axes (Figure 2a). The Gran Paradiso source population was at the center of the three axes of the population substructure. PC1 and PC2 explained similar amounts of variance (10.1% and 8.4%, respectively) indicating similar degrees of genetic differentiation along the three axes representing the substructure of Alpine ibex described above. At the extremes of two axes were the Weisshorn and Cape au Moine populations (pairwise *F*_ST_ = 0.10; Fig. S6). Both populations were founded almost exclusively from a single primary population (Pleureur and Brienzer Rothorn, respectively). The pairwise *F*_ST_ between Pleureur and Brienzer Rothorn was 0.08. The two populations Schwarz Mönch and Aletsch Bietschhorn (purple in Figure 2a) were close to the center of the PCA. In contrast to Weisshorn and Cape au Moine, these two populations were founded from multiple source populations (Biebach & Keller, 2009). Pairwise *F*_ST_ between Gran Paradiso and the reintroduced ibex populations ranged from 0.04 (Aletsch Bietschhorn) to 0.08 (Weisshorn and Cape au Moine; Figure S6).

A Bayesian clustering analysis using STRUCTURE (Pritchard et al., 2000) confirmed the grouping identified by the PCA and identified the mixed ancestry of individuals from the ab and sm populations (Fig. S7). The optimal *K* was *K* = 2 according to the delta *K* method (Evanno, Regnaut, & Goudet, 2005). The source population of all Alpine ibex (gp) was composed of multiple clusters. This is consistent with the fact that individuals of the source population were used to reintroduce all primary and secondary populations. Hence, clusters identified among reintroduced populations should also be represented in the source population.

The hierarchical AMOVA mirrored the results of PCA and STRUCTURE analysis and found significant differentiation among the groups (comprising the respective primary and secondary reintroduced populations) and among all individual populations (Table S3). However, no significant differentiation (*p *=* *.26) was found between source and reintroduced populations.

### 3.3 Genetic diversity in populations

**Figure 3:**
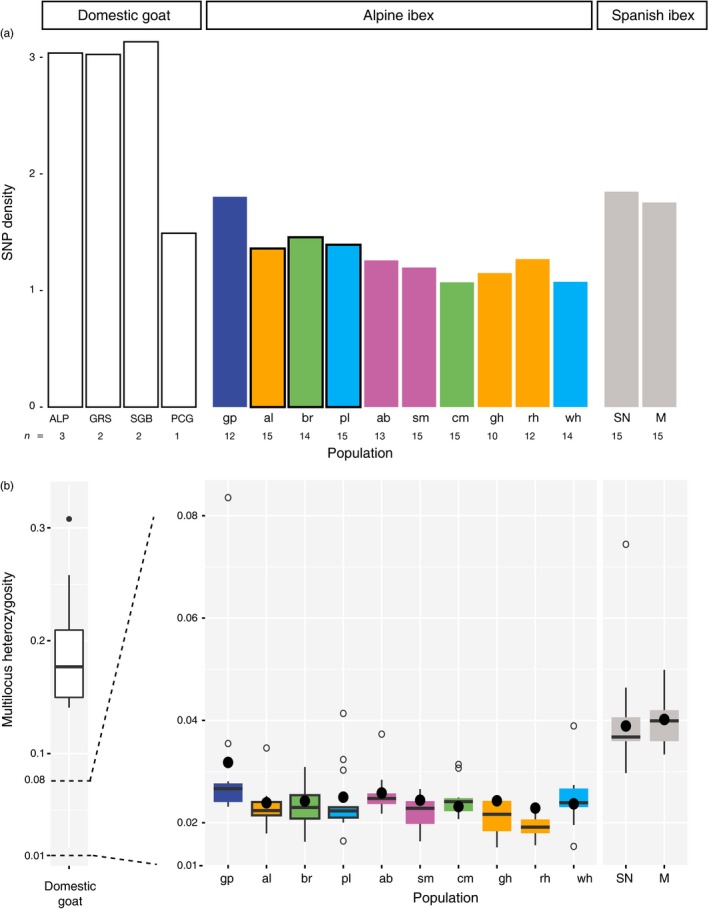
Genetic diversity estimates based on SNP density and multilocus heterozygosity. (a) The SNP density in SNPs per kilobase of sequence is shown for domestic goat breeds, Iberian, and Alpine ibex populations. Black outlines show the three primary reintroduced populations Albris (orange), Pleureur (light blue), and Brienzer Rothorn (green). Secondary reintroductions established from the primary reintroduced populations share the same color. Populations with mixed ancestry are shown in purple. (b) The individual multilocus heterozygosity is shown summarized per population (outliers are shown as empty circles). Filled circles represent the expected heterozygosity per population. Domestic goat breeds: ALP, Gemsfarbene Gebirgsziegen; GRS, Grison striped; SGB, St. Gallen booted; PCG, Peacock goat. Ibex populations abbreviated as in Figure 1a

**Figure 4:**
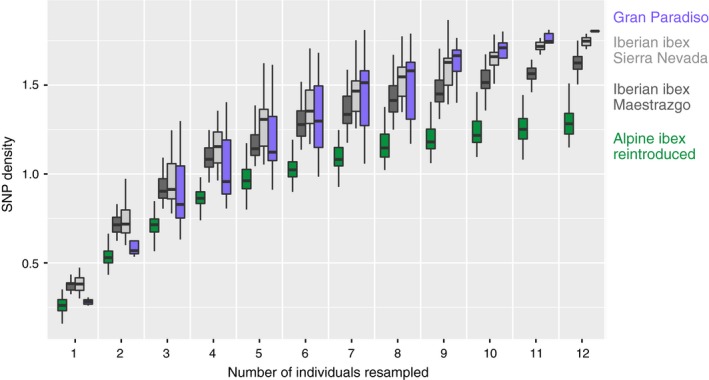
Estimates for total genetic diversity using resampling curves of Alpine ibex and Iberian ibex. Boxplots summarize 100 resampled SNP densities (SNP per kilobase of sequence) for sample sizes between 1 and 12. See Fig. S8A for resampling curves including domestic goat

The Gran Paradiso Alpine ibex population and the Iberian ibex populations give insights into levels of genetic diversity in populations of ibex that have experienced bottlenecks but not reintroductions. Comparing the genetic diversity of the Gran Paradiso population with the reintroduced populations gives insights into the impact of reintroduction bottlenecks. SNP density (SNPs per kilobase sequenced) was similar in the Gran Paradiso population and the two Iberian ibex populations. However, SNP density was lower in all reintroduced Alpine ibex populations (Figure 3). As estimates of genetic diversity can be affected by sampling effort, we also used a resampling procedure to compare genetic diversity among groups. We found higher SNP densities in the autochthonous Gran Paradiso population and Iberian ibex than in the pool of the reintroduced populations. In comparison, domestic goat had the highest SNP density (Fig. S8A). Based on resampling 12 individuals, we found a median SNP density of 1.3 SNPs per kilobase of sequence across all reintroduced Alpine ibex populations, of 1.6 and 1.7 SNPs/kb in the two autochthonous populations of Iberian ibex, and of 1.8 SNPs/kb in Alpine ibex from Gran Paradiso (Figure 4). Resampling curves of SNP densities estimated for individual populations confirmed higher SNP densities in primary than in secondary reintroduced populations (Fig. S8B).

Expected heterozygosity was highest in the two Iberian ibex populations (0.039 and 0.040 for Sierra Nevada and Maestrazgo, respectively), intermediate in Gran Paradiso (0.032) and lowest in the reintroduced populations (0.023–0.026; filled circles in Figure 3b and Table 1). Domestic goats had the highest expected heterozygosity.

### 3.4 Comparisons of effective population sizes

**Figure 5:**
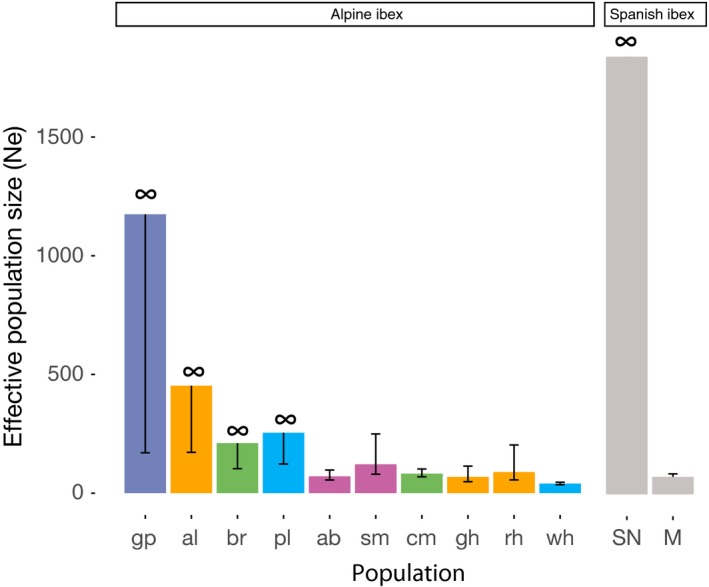
Estimates for the effective population size (*N*_*e*_) of different Alpine ibex and Iberian ibex populations using a minor allele frequency threshold of 0.1. Populations abbreviated as in Figure 1a. Whiskers show the confidence intervals of *N*_*e*_ estimates (infinite upper bounds are represented by symbols)

Estimates of effective population sizes revealed that all secondary reintroduced Alpine ibex populations had a *N*_*e*_ of 40–120. The Iberian ibex population Maestrazgo had a comparable *N*_*e*_ (Figure 5 and Table S4). The three primary reintroduced Alpine ibex populations had larger *N*_*e*_, in the range of 210–450. The Gran Paradiso and Sierra Nevada populations had *N*_*e*_ of ~1,000. Several *N*_*e*_ estimates in Figure 5 have infinite upper bounds, which may at least partly be explained by the small population sample sizes. However, all the *N*_*e*_ confidence intervals estimated for secondary reintroduced populations had finite upper bounds. The minor allele frequency (MAF) thresholds used for this analysis can have an impact on the estimates of Ne. The analyses above were performed with MAF > 0.1 (see Table S4 for *N*_*e*_ estimates based on different MAF thresholds). Note that Waples and Do (2008) showed that *N*_*e*_ estimations performed better for MAF > 0.1 than for lower thresholds (i.e., higher percentage of confidence intervals included the true Ne).

Although the upper bounds of the estimates were often infinite, comparisons of the *N*_*e*_ estimates of the secondary reintroduced populations with observed levels of genetic differentiation among populations suggest that the point estimates of *N*_*e*_ are reasonable. Assuming no differentiation at the time of reintroduction (i.e., assuming *F*_0_ = 0), pairwise *F*_ST_ is predicted to increase over time according to $1-F_{\mathrm{ST}}={\left(1-\frac{1}{2N_{e}}\right)}^{t}$ (Wright, 1969), where the increase in *F*_ST_ represents the increase in identity by descent due to genetic drift and *t* the generation time. Pairwise *F*_ST_ estimates between Gran Paradiso and the reintroduced populations were 0.04–0.08. The reintroductions were initiated 8–10 generations ago. *N*_*e*_ estimated from pairwise *F*_ST_ assumes equal *N*_*e*_ between populations (or one population with infinite *N*_*e*_). Although this assumption is unlikely to be true, harmonic mean *N*_*e*_ estimates based on pairwise *F*_ST_ values indicated a *N*_*e*_ of 120 in primary reintroduced populations (lower than estimated from LD) and a *N*_*e*_ of 60 in secondary reintroduced populations.

### 3.5 Individual‐based levels of recent inbreeding

**Figure 6:**
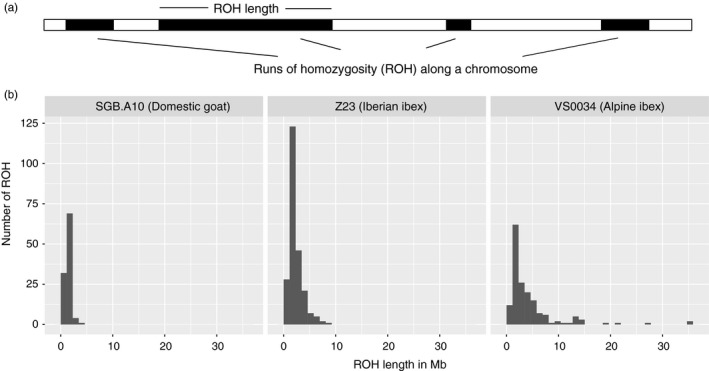
Runs of homozygosity in different species. (a) Schematic showing individual runs of homozygosity (ROH) identified in a chromosomal sequence. (b) Size distribution of ROH for three individuals with different total ROH lengths. The total ROH lengths are shown for a domestic goat (SGB.A10), an Iberian ibex (Z23), and an Alpine ibex (VS0034)

**Figure 7:**
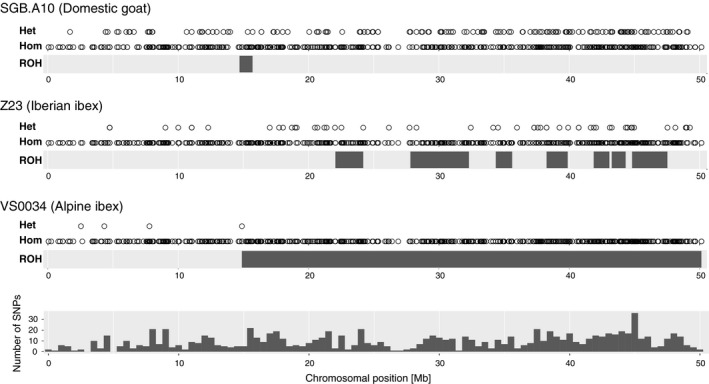
Runs of homozygosity detected in domestic goat (SGB.A10), Iberian ibex (Z23), and Alpine ibex (VS0034) along chromosome 26. For each individual, the genotype at each SNP is indicated as being either heterozygote (“Het”) or homozygote (“Hom”). The runs of homozygosity (ROH) are shown as black bars along the chromosome. The bottom panel shows the number of SNPs per 0.5 Mb along chromosome 26

**Figure 8:**
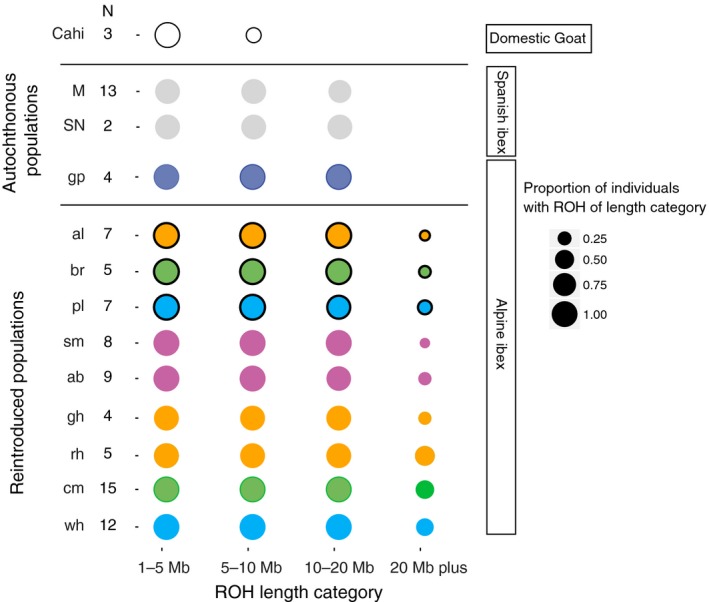
Tract length distribution of runs of homozygosity in autochthonous and reintroduced populations. Runs of homozygosity (ROH) in individuals show a range of different tract lengths. For each size category of runs of homozygosity (ROH) tract lengths, the proportion of sampled individuals in a population carrying such ROH tract lengths in the genome is shown. Black outlined circles show the three primary reintroduced populations Albris (orange), Pleureur (light blue), and Brienzer Rothorn (green). Secondary reintroductions established from the primary reintroduced populations share the same color. Populations with mixed ancestry are shown in purple. Populations abbreviated as in Figure 1a. *N*, sample size per population

We estimated inbreeding among ibex individuals using runs of homozygosity (ROH) along the genome (Figure 6). Using stringent filters, we predominantly identified ROH in regions of high SNP densities (Fig. S9). Long ROH indicate recent inbreeding (see Figure 7 for examples). Total lengths of all ROH including short ROH, which are not indicative of recent consanguinity, were largely identical among ibex populations (Fig. S10A). However, the total length of long ROH (>5 Mb), which are indicative of recent inbreeding, varied among populations (Fig. S10B). In Alpine ibex, we found that the population median of total length of long ROH (>5 Mb) was at least 216 Mb (Gran Paradiso) and covered at least 9% of the autosomal genome (Fig. S10B). The Iberian ibex populations Sierra Nevada and Maestrazgo had median total length of long ROH (>5 Mb) 228 Mb (*n *=* *3) and 179 Mb, respectively. In comparison, the total length of ROH (>5 Mb) was less than 10 Mb in all domestic goat. Reintroduced populations had the longest total length of ROH (Fig. S10B), and ROH longer than 20 and 30 Mb were only observed in reintroduced populations (Figure 8). In domestic goat, no ROH longer than 10 Mb was observed (Figure 8).

Finally, we correlated alternative estimates of inbreeding with ROH. The molecular inbreeding coefficient FROH_10,000_ is defined as the total length of ROH longer than 10 Mb divided by the autosomal genome length (Purfield et al., 2012). Multilocus homozygosity and FROH_10,000_ were positively correlated across individuals (*R* ^2^ = .29, *p *<* *.0001, *n *=* *92) (Fig. S11). Biebach and Keller (2010) used population‐specific *F*_ST_ to estimate population inbreeding. The *F*_ST_ used for these estimates reflected the differentiation of the focal population compared to all other populations. We found a weak but significant positive correlation between FROH_10,000_ and population inbreeding estimated based on *F*_ST_ (*R* ^2^ = .047, *p *=* *.04, Fig. S12).

## 4 DISCUSSION

Sustainable species conservation depends on understanding the genetic consequences of management strategies. Here, we described a systematic genomic survey and population genetic analysis of a species that was successfully reintroduced into a fragmented alpine habitat. We compared the autochthonous source population of all reintroduced Alpine ibex populations, several reintroduced Alpine ibex populations, two autochthonous populations of a closely related species and several breeds of the related domestic goat. The systematic sampling and high‐density markers allowed investigating the effects of two critical stages of a species reintroduction (i.e., the selection of individuals for transfers and the impact of demography on genetic diversity after the reintroductions) on genomewide polymorphism.

### 4.1 The application of RAD‐seq to recapitulate demographic processes in a reintroduced species

Our analyses were based on high‐throughput RAD‐seq genotyping. High‐throughput genotyping such as RAD‐seq and other barcoded next‐generation sequencing techniques can introduce potential biases in the genotypic dataset. We performed a series of analyses to identify issues arising specifically from barcoded sequencing libraries that combine a large number of individuals of potentially different DNA quality. We found that uneven coverage among individuals (and populations) can introduce biases if these are not properly controlled for. For example, sequencing coverage was inversely correlated with genotype missingness (data not shown). Hence, we required a minimum coverage for any individual to be included in the analyses. We chose SNP density as a measure of genetic diversity rather than raw SNP counts to control for remaining variation in per‐individual coverage. Variation in the proportion of genotyped markers (i.e., genotyping rate) can influence measures of heterozygosity and population differentiation (e.g., Fig. S3). Therefore, we applied strict missingness filters to include only the best‐sequenced SNPs in such analyses. We also applied strict filters for marker density to produce conservative estimates of runs of homozygosity (ROH). Ascertainment bias may further be introduced by the fact that reads were aligned to a single reference genome from the domestic goat. However, we found that reads obtained from different species aligned nearly equally well to the reference genome. The ascertainment bias from aligning to the goat genome should therefore be small. Interestingly, the alignment rates of sequences obtained from domestic goat were lower on average than sequences obtained from ibex species. This is despite the fact that the reference genome was built from a domestic goat. The lower alignment rates of domestic goats are most likely explained by the substantial genetic diversity among domestic goat (samples representing Swiss breeds mapped to reference built from Chinese breed).

High‐throughput sequencing protocols for reduced representation libraries such as RAD‐seq include PCR cycles to amplify the small pool of primary DNA fragments. PCR produces duplicate fragments, which can be retained in the final pool of sequenced fragments. We found indeed PCR duplicates among the reads obtained from each individual. The proportion of duplicates was in the range of typical RAD‐seq studies (for a review see Andrews, Good, Miller, Luikart, & Hohenlohe, 2016). PCR duplicates should not lead to systematic (i.e., genomewide) genotyping errors. However, PCR duplicates can be an issue if high genotyping accuracy is required at individual loci (e.g., for genomewide association studies). As our analyses were based on genomewide estimates of population genetic parameters, we retained PCR duplicates in our analyses. We found that the presence of PCR duplicates had no discernable impact on estimates of population differentiation (see Fig. S5).

### 4.2 The impact of demography on reintroduced populations

The primary aim of our study was to quantify the loss of genomewide diversity due to the reintroduction of Alpine ibex from a single source population (Gran Paradiso). We found strong evidence for within‐species substructure that reflects the reintroduction strategy. At the time of the first reintroductions in the early 20th century, there was likely virtually no substructure among Alpine ibex as the reintroductions were initiated from a single source population. Hence, the observed genetic differentiation among reintroduced populations was the likely result of genetic drift over the past 8–10 generations. The three primary reintroduced populations in Switzerland showed considerable genetic differentiation. The repeated founding of secondary populations mostly from a single primary population exacerbated the genetic differentiation from the Gran Paradiso source population. Estimates for genetic structure among reintroduced populations based on microsatellite markers (Biebach & Keller, 2009) were very similar to our own estimates. In addition, our data clearly identified a stepwise increase in genetic substructure with subsequent rounds of reintroductions. This indicates that gene flow was very low or absent among the three main genetic groups composed of three primary reintroduced populations. In addition to strong founder effects of the reintroductions, gene flow was likely low between the primary and their respective secondary populations, once translocations of animals stopped. Reduced gene flow may be a concern for the long‐term viability of Alpine ibex, in particular because genetic diversity was low in most populations.

Our genomewide analyses showed that reintroduced populations of Alpine ibex had lower SNP densities and heterozygosity than the Gran Paradiso source population. Assuming that eight generations elapsed between the reintroductions and our sampling, the reduction in expected heterozygosity of 15%–25% between Gran Paradiso and the reintroduced populations corresponds to a harmonic mean *N*_*e*_ of 15–25 individuals (following the equation *H*_*t*+1_ = (1 − 1/2*N*_*e*_)*H_*t*_). Although there was considerable variation in estimates of *N*_*e*_ based on loss of expected heterozygosity, *F*_ST_ and LD, all methods suggest that the secondary reintroduced populations had effective population sizes of 120 individuals or fewer. Frankham, Bradshaw, and Brook (2014) suggested that a minimum *N*_*e*_ of 100 and 1,000 was necessary for the short‐term survival and long‐term survival of a population, respectively. For Alpine ibex, a key piece of information would be knowledge about historic, prebottleneck *N*_*e*_. Historic levels of *N*_*e*_ would indicate how the discontinuous nature of the alpine habitat impacted gene flow among populations, without anthropogenic effects such as habitat destruction and overhunting.

### 4.3 Comparative population genomic analyses of European ibex and domestic goat

Alpine ibex suffered a unique population bottleneck compared to other ibex species due to the near extinction in the 19th century. However, the Iberian ibex occupy an alpine habitat that is similarly fragmented by topography. Both autochthonous populations of Iberian ibex and the source population of Alpine ibex were confronted with strong barriers to dispersal. At high population densities, some barriers might be overcome due to high dispersal pressure. Such dispersal may lead to the colonization of new territories or to population expansion (Refoyo, Olmedo, Polo, Fandos, & Muñoz, 2015; Refoyo et al., 2016). However, dispersal barriers between the two Iberian ibex populations seem to be effective and demographic bottlenecks are evident leading to a high differentiation from each other. The genetic differentiation is higher than the most differentiated Alpine ibex populations (pairwise *F*_ST_ = 0.39 vs. *F*_ST_ = 0.12). The two Iberian ibex populations experienced each two distinct bottlenecks, and the two populations have been isolated for about 25 generations since the drastic census reductions in the early 19th century (Samer Angelone‐Alasaad, pers. comm.).

Domestic goat exhibited the highest levels of genetic diversity. This may seem surprising because most domesticated plant and animal species suffered from considerable losses in genetic diversity (e.g., Blackman et al., 2011; Renaut & Rieseberg, 2015; Ross‐Ibarra, Morrell, & Gaut, 2007; Zeder, Emshwiller, Smith, & Bradley, 2006), deleterious mutations accumulation (e.g., sunflower: Renaut & Rieseberg, 2015; dogs: Marsden et al., 2016), and inbreeding (e.g., Marsden et al., 2016). However, the domestication of goats was likely more complex than in most other domesticated animals as the extant domestic goat may have multiple origins of domestication (Luikart et al., 2001). In addition, neither of the two ibex species under study are direct sister species of the domestic goat. Genetic diversity data of the wild sister species of domestic goat, bezoar (*Capra aegagrus*), may provide further insights into the origins of the domestic goat diversity.

### 4.4 Genetic diversity in autochthonous ibex populations

All populations in this study, including Iberian ibex populations, suffered from bottlenecks. Nevertheless, we observed that the levels of genetic diversity estimated by SNP density and multilocus heterozygosity were highest in the Iberian ibex populations and the Gran Paradiso Alpine ibex population, and lowest in the reintroduced Alpine ibex populations. The similarly high levels of diversity in the two Iberian populations were not predicted from knowledge about the demographic history of these two populations. The Maestrazgo population suffered from a population bottleneck an order of magnitude stronger than the Sierra Nevada population (~30 vs. hundreds of individuals, Couturier, 1962). However, gene flow (including not recorded translocations) might have enhanced genetic diversity of the Maestrazgo population. The population Gran Paradiso went through a bottleneck of <100 individuals at the beginning of the 18th century and a second, much less severe bottleneck mid‐20th century with *N* ~1,000. Although SNP density was comparable between Gran Paradiso and the Iberian ibex, expected heterozygosity was considerably lower in Gran Paradiso. This discrepancy may be explained by a higher proportion of low‐frequency polymorphism in the Gran Paradiso (Fig. S13). Population genetics theory predicts that severe bottlenecks over few generations can have a similar impact on genetic diversity to mild bottlenecks over longer periods. This may explain why we observed levels of genetic diversity among the Iberian ibex and Gran Paradiso populations that did not match predictions made from demographic information about bottlenecks. Furthermore, census data reported for specific bottlenecks can be a poor predictor of effective levels of genetic diversity. This may be due to the fact that the reported data were inaccurate or that additional demographic information, such as the number of reproducing individuals and the occurrence of immigration, is needed.

Autochthonous populations (Gran Paradiso and Iberian ibex) had consistently higher genetic diversity than reintroduced populations. One Gran Paradiso individual showed extraordinarily high levels of heterozygosity (>0.08, Figure 3b), which increased the SNP density of the Gran Paradiso population. However, even after excluding the individual, SNP density was still higher than the pool of reintroduced populations (Fig. S8C). This suggests that reintroductions to the Swiss Alps led to consistent losses in genetic diversity. A previous study based on microsatellites was unable to resolve these differences between the source and the pool of reintroduced populations (Biebach & Keller, 2009).

### 4.5 Runs of homozygosity to accurately assess levels of recent inbreeding

A major concern for the management of reintroduced species is that the reduced levels of genetic diversity cause harmful inbreeding. However, detecting early inbreeding in natural populations can be challenging. Traditionally, inbreeding was estimated using pedigree data (which are difficult to obtain from wild populations) or multilocus heterozygosity. The affordability of RAD‐seq or genotyping by sequencing led to the implementation of genomewide inference in a number of recent conservation studies despite considerable challenges (Shafer et al., 2015). Genomewide markers allow powerful estimates of inbreeding based on runs of homozygosity (ROH), which have so far mostly been performed in studies of human and domestic animals (e.g., Lencz et al., 2007; McQuillan et al., 2008; Pemberton et al., 2012; Purfield et al., 2012). A key requirement for ROH estimates is knowledge of physical marker positions. Such information is often only available if a reference genome of the same or a closely related species exists. The rapid increase in the number of available genome sequences from wild species should facilitate ROH analyses in many species of conservation concern. Although pedigree, ROH‐based inbreeding estimates, and multilocus homozygosity are often correlated (e.g., Purfield et al., 2012), the distributions of ROH tract lengths yield additional information about when inbreeding occurred in the ancestry of individuals. Uncertainty in pedigree estimates about recent inbreeding stems from the fact that the same pedigree relatedness can mask variable levels of identity by descent (Kardos et al., 2015; McQuillan et al., 2008). Furthermore, ROH‐based estimates of inbreeding can be performed even if only a small number of individuals are available. Short ROH in a population indicate recurrent breeding among distant relatives and historically small *N*_*e*_, but not necessarily recent inbreeding (Pemberton et al., 2012). This situation is typically found in outbred human individuals, where ROH tracts up to 4 Mb are relatively common (McQuillan et al., 2008). ROH tracts longer than 5 Mb are generally interpreted as a shared maternal and paternal ancestor in the preceding six generations, and ROH tracts in the tens of Mb indicate a high degree of relatedness among the parents. ROH tracts longer than 20 Mb were reported in cattle breeds, where artificial selection and artificial insemination lead to considerable inbreeding (e.g., Purfield et al., 2012).

The number of SNPs included in the genotyping assay can have an effect on ROH‐based inbreeding estimates. The effect is stronger in individuals with short ROH. In our study, we used 41,907 SNPs for ROH calculations. This number is comparable to 50k SNP chips that are frequently used for instance in studies of ROH in cattle. As RAD‐seq or SNP‐chip genotyping identifies only polymorphisms in a subset of the genome, ROH are not reliably detectable below a certain tract length. To avoid biases in ROH‐based estimates due to incomplete marker information, we excluded ROH shorter than 1 Mb, although estimates up to 5 Mb may suffer from ascertainment biases (Purfield et al., 2012). Furthermore, we only considered regions for ROH with above‐average SNP densities. Therefore, we may have underestimated the extent of ROH in certain genomic regions (see Fig. S9).

All individuals in our study were found to have ROH up to 5 Mb. The total length of ROH was similar among all Alpine and Iberian ibex populations and comparable to estimates based on whole genome sequencing in other mammals. In inbred Gorilla populations, ROHs of 2.5–10 Mb covered between 8% and 20% of the total genome (Xue et al., 2015). ROH tracts of the same length category covered 14%–17% of the total autosomal genome length in the two ibex species. ROH tracts longer than 20 Mb were only observed in the reintroduced Alpine ibex populations. Hence, recent inbreeding was more frequent in the reintroduced populations than the Gran Paradiso source population. We observed individual ROH‐based inbreeding estimates (FROH_10,000_) up to 0.1. Values in this range were shown to correspond to pedigree inbreeding estimates of matings among half sibs (pedigree *F* > 0.125; Purfield et al., 2012). The high level of inbreeding in reintroduced Alpine ibex populations suggests that inbreeding depression may be expressed. Indeed, a study of Alpine ibex in the Gran Paradiso revealed inbreeding depression in several fitness‐related traits (Brambilla et al., 2015).

### 4.6 Applying genomic surveys to assess the genetic status of reintroduced populations

Many endangered species suffered from population bottlenecks in their recent history. Species with a significantly diminished distribution range can be conserved by population reintroductions. However, population reintroductions repeatedly failed to meaningfully extend lost species ranges (Armstrong & Seddon, 2008). Despite the large number of population reintroductions, few studies investigated the long‐term genetic consequences of reintroductions. Such consequences critically include the potential loss of genetic diversity and increased inbreeding levels due to genetic bottlenecks. Following bottlenecks, populations may have a reduced potential to adapt to a changing environment or resistance to new diseases (O'Brien & Evermann, 1988). The population genetic consequences of bottlenecks were convincingly demonstrated using microsatellites and mitochondrial markers in multiple species (e.g., Biebach & Keller, 2009, 2010; Diefenbach et al., 2015; Jamieson, 2011; Strzała et al., 2015). However, only few studies included genetic data from the source population and monitoring data of the entire population reintroduction history. Using genomewide inference, our study showed that population reintroductions of the Alpine ibex lowered genetic diversity and increased recent inbreeding. Both Iberian ibex populations and the Alpine ibex source population maintained higher levels of genetic diversity and lower levels of recent inbreeding. However, our estimates of genetic diversity in these autochthonous populations did not correspond to census information on historic population bottlenecks. Census information can be a poor predictor of effective levels of genetic diversity, because reported data may be inaccurate and not fully informative about demography (e.g., migration and sex ratios). Hence, genomic surveys serve as an important monitoring tool even if census data are available. Consideration of the population genomic consequences of conservation efforts should become an integral component of managing reintroductions.

### 4.7 Management recommendations

Our study identified low genetic diversity and high inbreeding levels in reintroduced populations of Alpine ibex. Effects were even more pronounced in secondary vs. primary reintroduced populations. Low genetic diversity might reduce fitness in the long‐term due to reduced evolutionary potential (Allendorf et al., 2012; Biebach, Leigh, Sluzek, & Keller, 2016). Inbreeding levels were in a range where inbreeding is expected to impact fitness (e.g., Huisman, Kruuk, Ellis, Clutton‐Brock, & Pemberton, 2016; Keller & Waller, 2002). Thus, existing Alpine ibex populations would benefit from introducing individuals from genetically differentiated populations to increase genetic variation and reduce inbreeding. Such measures may be necessary to preserve the long‐term survival of Alpine ibex. Results of this study show that secondary reintroductions from a sole primary reintroduced population should be avoided. Instead, new populations should be founded with individuals from the source population (GP) or from multiple primary reintroduced populations. This is in line with a previous study with microsatellites where the degree of admixture of the founder group had a higher impact on genetic variation than the number of founders (Biebach & Keller, 2012).

The re‐establishment of Alpine ibex across the Alps was one of the most successful species reintroductions. Alpine ibex recovered from about 100 individuals to ca. 50,000 individuals across the Alps. Our study showed that although demographic risks are currently minimal, genetic (i.e., inbreeding) risks due to the reintroduction history persist. Therefore, future reintroductions should be genetically monitored even after the successful establishment of a population, with a particular focus on early signs of inbreeding. Such monitoring would ensure the early detection of genetic problems of endangered species and would allow initiating the necessary management actions.

## DATA ARCHIVING STATEMENT

The SNP Data set is available from the Dryad Digital Repository: https://doi.org/10.5061/dryad.n276d.

## References

[eva12490-bib-0001] Alasaad, S. , Biebach, I. , Grossen, C. , Soriguer, R. C. , Pérez, J. M. , & Keller, L. F. (2012). Microsatellite‐based genotyping of MHC class II DRB1 gene in Iberian and Alpine ibex. European Journal of Wildlife Research, 58, 743–748.

[eva12490-bib-0002] Allendorf, F. W. , Hohenlohe, P. A. , & Luikart, G. (2010). Genomics and the future of conservation genetics. Nature Reviews Genetics, 11, 697–709. 10.1038/nrg2844 20847747

[eva12490-bib-0003] Allendorf, F. W. , Luikart, G. , & Aitken, S. N. (2012). Conservation and the genetics of populations, 2nd ed., Hoboken, NJ: Wiley‐Blackwell.

[eva12490-bib-0004] Amills, M. , Jimenez, N. , Jordana, J. , Riccardi, A. , Fernandez‐Arias, A. , Guiral, J. , … Sànchez, A. (2004). Low diversity in the major histocompatibility complex class II DRB1 gene of the Spanish ibex, *Capra pyrenaica* . Heredity, 93, 266–272. 15241456 10.1038/sj.hdy.6800499

[eva12490-bib-0005] Andrews, K. R. , Good, J. M. , Miller, M. R. , Luikart, G. , & Hohenlohe, P. A. (2016). Harnessing the power of RADseq for ecological and evolutionary genomics. Nature Publishing Group, 17, 81–92. 10.1038/nrg.2015.28 PMC4823021 26729255

[eva12490-bib-0006] Armstrong, D. P. , & Seddon, P. J. (2008). Directions in reintroduction biology. Trends in Ecology & Evolution, 23, 20–25. 18160175 10.1016/j.tree.2007.10.003

[eva12490-bib-0007] Biebach, I. , & Keller, L. F. (2009). A strong genetic footprint of the re‐introduction history of Alpine ibex (*Capra ibex ibex*). Molecular Ecology, 18, 5046–5058. 19912536 10.1111/j.1365-294X.2009.04420.x

[eva12490-bib-0008] Biebach, I. , & Keller, L. F. (2010). Inbreeding in reintroduced populations: the effects of early reintroduction history and contemporary processes. Conservation Genetics, 11, 527–538.

[eva12490-bib-0009] Biebach, I. , & Keller, L. F. (2012). Genetic variation depends more on admixture than number of founders in reintroduced Alpine ibex populations. Biological Conservation, 147, 197–203.

[eva12490-bib-0010] Biebach, I. , Leigh, D. M. , Sluzek, K. , & Keller, L. F. (2016). Genetic issues in reintroduction In Jachowski D. S., Millspaugh J. J., Angermeier P. L., & Slotow R. (Eds.), Reintroduction of fish and wildlife populations (pp. 149–183). Oakland, CA: University of California Press.

[eva12490-bib-0011] Blackman, B. K. , Scascitelli, M. , Kane, N. C. , Luton, H. H. , Rasmussen, D. A. , Bye, R. A. , … Rieseberg, L. H. (2011). Sunflower domestication alleles support single domestication center in eastern North America. Proceedings of the National Academy of Sciences, 108, 14360–14365. 10.1073/pnas.1104853108 PMC3161615 21844335

[eva12490-bib-0012] Bolger, A. M. , Lohse, M. , & Usadel, B. (2014). Trimmomatic: A flexible trimmer for Illumina sequence data. Bioinformatics, 30, 2114–2120. 24695404 10.1093/bioinformatics/btu170 PMC4103590

[eva12490-bib-0013] Brambilla, A. , Biebach, I. , Bassano, B. , Bogliani, G. , & von Hardenberg, A. (2015). Direct and indirect causal effects of heterozygosity on fitness‐related traits in Alpine ibex. Proceedings of the Royal Society B‐Biological Sciences, 282, 20141873. 10.1098/rspb.2014.1873 PMC4262168 25392468

[eva12490-bib-0014] Broman, K. W. , & Weber, J. L. (1999). Long homozygous chromosomal segments in reference families from the centre d'Etude du polymorphisme humain. American Journal of Human Genetics, 65, 1493–1500. 10577902 10.1086/302661 PMC1288359

[eva12490-bib-0015] Couturier, M. (1962). Le Bouquetin des Alpes. Grenoble, France.

[eva12490-bib-0016] Danecek, P. , Auton, A. , Abecasis, G. , Albers, C. A. , Banks, E. , DePristo, M. A. , … 1000 Genomes Project Analysis Group . (2011). The variant call format and VCFtools. Bioinformatics, 27, 2156–2158. 21653522 10.1093/bioinformatics/btr330 PMC3137218

[eva12490-bib-0017] DePristo, M. A. , Banks, E. , Poplin, R. , Garimella, K. V. , Maguire, J. R. , Hartl, C. , … Daly, M. J. (2011). A framework for variation discovery and genotyping using next‐generation DNA sequencing data. Nature Genetics, 43, 491–498. 21478889 10.1038/ng.806 PMC3083463

[eva12490-bib-0018] Diefenbach, D. , Hansen, L. , Bohling, J. , & Miller Butterworth, C. (2015). Population and genetic outcomes 20 years after reintroducing bobcats (*Lynx rufus*) to Cumberland Island, Georgia USA. Ecology and Evolution, 5, 4885–4895. 26640668 10.1002/ece3.1750 PMC4662311

[eva12490-bib-0019] Do, C. , Waples, R. S. , Peel, D. , Macbeth, G. M. , Tillett, B. J. , & Ovenden, J. R. (2014). NeEstimatorv2: Re‐implementation of software for the estimation of contemporary effective population size (Ne) from genetic data. Molecular Ecology Resources, 14, 209–214. 23992227 10.1111/1755-0998.12157

[eva12490-bib-0020] Dong, Y. , Xie, M. , Jiang, Y. , Xiao, N. , Du, X. , Zhang, W. , … Wang, W. (2012). Sequencing and automated whole‐genome optical mapping of the genome of a domestic goat (*Capra hircus*). Nature Biotechnology, 31, 135–141. 10.1038/nbt.2478 23263233

[eva12490-bib-0021] Evanno, G. , Regnaut, S. , & Goudet, J. (2005). Detecting the number of clusters of individuals using the software STRUCTURE: A simulation study. Molecular Ecology, 14(8), 2611–2620. https://doi.org/10.1111/j.1365-294X.2005.02553.x 15969739 10.1111/j.1365-294X.2005.02553.x

[eva12490-bib-0022] Frankham, R. , Bradshaw, C. J. A. , & Brook, B. W. (2014). Genetics in conservation management: Revised recommendations for the 50/500 rules, Red List criteria and population viability analyses. Biological Conservation, 170, 56–63.

[eva12490-bib-0023] Giacometti M. (Ed.) (2006). Von Königen und Wilderern: Die Rettung und Wiederansiedlung des Alpensteinbockes. Bern: Salm Verlag Wohlen.

[eva12490-bib-0024] Grossen, C. , Keller, L. , Biebach, I. , International Goat Genome Consortium , & Croll, D. (2014). Introgression from domestic goat generated variation at the major histocompatibility complex of Alpine ibex. PLoS Genetics, 10, e1004438. 24945814 10.1371/journal.pgen.1004438 PMC4063738

[eva12490-bib-0025] Grubb, P. (2005). Order artiodactyla In Wilson D. E., & Reeder D. M. (Eds.), Mammal species of the world: A taxonomic and geographic reference (pp. 637–722). Baltimore, USA: The Johns Hopkins University Press.

[eva12490-bib-0026] Hoffman, J. I. , Simpson, F. , David, P. , Rijks, J. M. , Kuiken, T. , Thorne, M. A. S. , … Dasmahapatra, K. K. (2014). High‐throughput sequencing reveals inbreeding depression in a natural population. Proceedings of the National Academy of Sciences, 111, 3775–3780. 10.1073/pnas.1318945111 PMC3956162 24586051

[eva12490-bib-0027] Huisman, J. , Kruuk, L. E. B. , Ellis, P. A. , Clutton‐Brock, T. , & Pemberton, J. M. (2016). Inbreeding depression across the lifespan in a wild mammal population. Proceedings of the National Academy of Sciences, 113, 3585–3590. 10.1073/pnas.1518046113 PMC4822623 26979959

[eva12490-bib-0028] Jamieson, I. G. (2011). Founder effects, inbreeding, and loss of genetic diversity in four avian reintroduction programs. Conservation Biology, 25, 115–123. 20825445 10.1111/j.1523-1739.2010.01574.x

[eva12490-bib-0029] Jamieson, I. G. , & Lacy, R. C. (2012). Managing genetic issues in reintroduction biology In Ewen J. G., Armstrong D. P., Parker K. A., & Seddon P. J. (Eds.), Reintroduction biology: Integrating science and management (pp. 441–475). Oxford: Wiley‐Blackwell.

[eva12490-bib-0030] Kardos, M. , Luikart, G. , & Allendorf, F. W. (2015). Measuring individual inbreeding in the age of genomics: Marker‐based measures are better than pedigrees. Heredity, 115, 63–72. 26059970 10.1038/hdy.2015.17 PMC4815495

[eva12490-bib-0031] Kardos, M. , Taylor, H. R. , Ellegren, H. , Luikart, G. , & Allendorf, F. W. (2016). Genomics advances the study of inbreeding depression in the wild. Evolutionary Applications, 9, 1205–1218. 27877200 10.1111/eva.12414 PMC5108213

[eva12490-bib-0032] Keller, L. (1998). Inbreeding and its fitness effects in an insular population of song sparrows (*Melospiza melodia*). Evolution, 52, 240–250. 28568167 10.1111/j.1558-5646.1998.tb05157.x

[eva12490-bib-0033] Keller, L. , Arcese, P. , Smith, J. , Hochachka, W. M. , & Stearns, S. C. (1994). Selection against inbred song sparrows during a natural‐population bottleneck. Nature, 372, 356–357. 7969492 10.1038/372356a0

[eva12490-bib-0034] Keller, L. , Marr, A. B. , & Reid, J. M. (2006). The genetic consequences of small population size: Inbreeding and loss of genetic variation In Smith J., Keller L. F., Marr A. B., & Arcese P. (Eds.), Conservation and biology of small populations (pp. 113–137). New York, NY: Oxford University Press.

[eva12490-bib-0035] Keller, M. C. , Visscher, P. M. , & Goddard, M. E. (2011). Quantification of inbreeding due to distant ancestors and its detection using dense single nucleotide polymorphism data. Genetics, 189, 237–249. 21705750 10.1534/genetics.111.130922 PMC3176119

[eva12490-bib-0036] Keller, L. , & Waller, D. (2002). Inbreeding effects in wild populations. Trends in Ecology & Evolution, 17, 230–241.

[eva12490-bib-0037] Langmead, B. , & Salzberg, S. L. (2012). Fast gapped‐read alignment with Bowtie 2. Nature Methods, 9, 357–359. 22388286 10.1038/nmeth.1923 PMC3322381

[eva12490-bib-0038] Lencz, T. , Lambert, C. , DeRosse, P. , Burdick, K. E. , Morgan, T. V. , Kane, J. M. , … Malhotra, A. K. (2007). Runs of homozygosity reveal highly penetrant recessive loci in schizophrenia. Proceedings of the National Academy of Sciences, 104, 19942–19947. 10.1073/pnas.0710021104 PMC2148402 18077426

[eva12490-bib-0039] Luikart, G. , Gielly, L. , Excoffier, L. , Vigne, J. D. , Bouvet, J. , & Taberlet, P. (2001). Multiple maternal origins and weak phylogeographic structure in domestic goats. Proceedings of the National Academy of Sciences, 98, 5927–5932. 10.1073/pnas.091591198 PMC33315 11344314

[eva12490-bib-0040] Manceau, V. , Crampe, J.‐P. , Boursot, P. , & Taberlet, P. (1999). Identification of evolutionary significant units in the Spanish wild goat, *Capra pyrenaica* (Mammalia, Artiodactyla). Animal Conservation, 2, 33–39.

[eva12490-bib-0041] Marsden, C. D. , Ortega‐Del Vecchyo, D. , O'Brien, D. P. , Taylor, J. F. , Ramirez, O. , Vilà, C. , … Lohmueller, K. E. (2016). Bottlenecks and selective sweeps during domestication have increased deleterious genetic variation in dogs. Proceedings of the National Academy of Sciences, 113, 152–157. 10.1073/pnas.1512501113 PMC4711855 26699508

[eva12490-bib-0042] McKenna, A. , Hanna, M. , Banks, E. , Sivachenko, A. , Cibulskis, K. , Kernytsky, A. , … DePristo, M. A. (2010). The genome analysis toolkit: A MapReduce framework for analyzing next‐generation DNA sequencing data. Genome Research, 20, 1297–1303. 20644199 10.1101/gr.107524.110 PMC2928508

[eva12490-bib-0043] McQuillan, R. , Leutenegger, A.‐L. , Abdel‐Rahman, R. , Franklin, C. S. , Pericic, M. , Barac‐Lauc, L. , … Wilson, J. F. (2008). Runs of homozygosity in European populations. The American Journal of Human Genetics, 83, 359–372. 18760389 10.1016/j.ajhg.2008.08.007 PMC2556426

[eva12490-bib-0044] Nei, M. (1973). Analysis of gene diversity in subdivided populations. Proceedings of the National Academy of Sciences, 70, 3321–3323. 10.1073/pnas.70.12.3321 PMC427228 4519626

[eva12490-bib-0045] O'Brien, S. J. , & Evermann, J. F. (1988). Interactive influence of infectious disease and genetic diversity in natural populations. Trends in Ecology & Evolution, 3, 254–259. 21227241 10.1016/0169-5347(88)90058-4 PMC7134056

[eva12490-bib-0046] Overall, A. , Byrne, K. , Pilkington, J. , & Pemberton, J. (2005). Heterozygosity, inbreeding and neonatal traits in Soay sheep on St Kilda. Molecular Ecology, 14, 3383–3393. 16156810 10.1111/j.1365-294X.2005.02682.x

[eva12490-bib-0047] Pemberton, T. J. , Absher, D. , Feldman, M. W. , Myers, R. M. , Rosenberg, N. A. , & Li, J. Z. (2012). Genomic patterns of homozygosity in worldwide human populations. American Journal of Human Genetics, 91, 275–292. 22883143 10.1016/j.ajhg.2012.06.014 PMC3415543

[eva12490-bib-0048] Perez, J. , Granados, J. , Soriguer, R. , Fandos, P. , Marquez, F. , & Crampe, J. (2002). Distribution, status and conservation problems of the Spanish Ibex, *Capra pyrenaica* (Mammalia : Artiodactyla). Mammal Review, 32, 26–39.

[eva12490-bib-0049] Pidancier, N. , Jordan, S. , Luikart, G. , & Taberlet, P. (2006). Evolutionary history of the genus *Capra* (Mammalia, Artiodactyla): Discordance between mitochondrial DNA and Y‐chromosome phylogenies. Molecular Phylogenetics and Evolution, 40, 739–749. 16757184 10.1016/j.ympev.2006.04.002

[eva12490-bib-0050] Pritchard, J. , Stephens, M. , & Donnelly, P. (2000). Inference of population structure using multilocus genotype data. Genetics, 155, 945–959. 10835412 10.1093/genetics/155.2.945 PMC1461096

[eva12490-bib-0051] Purcell, S. , Neale, B. , Todd‐Brown, K. , Thomas, L. , Ferreira, M. A. R. , Bender, D. , … Sham, P. C. (2007). PLINK: A tool set for whole‐genome association and population‐based linkage analyses. The American Journal of Human Genetics, 81, 559–575. 17701901 10.1086/519795 PMC1950838

[eva12490-bib-0052] Purfield, D. C. , Berry, D. P. , McParland, S. , & Bradley, D. G. (2012). Runs of homozygosity and population history in cattle. BMC Genetics, 13, 70–80. 22888858 10.1186/1471-2156-13-70 PMC3502433

[eva12490-bib-0053] Refoyo, P. , Olmedo, C. , & Muñoz, B. (2016). Space use of a reintroduced population of Iberian ibex (*Capra pyrenaica*) in a protected natural area. Canadian Journal of Zoology, 94, 181–189.

[eva12490-bib-0054] Refoyo, P. , Olmedo, C. , Polo, I. , Fandos, P. , & Muñoz, B. (2015). Demographic trends of a reintroduced Iberian ibex *Capra pyrenaica* victoriae population in central Spain. Mammalia, 79, 139–145.

[eva12490-bib-0055] Reid, J. , Arcese, P. , & Keller, L. (2003). Inbreeding depresses immune response in song sparrows (*Melospiza melodia*): Direct and inter‐generational effects. Proceedings of the Royal Society B‐Biological Sciences, 270, 2151–2157. 10.1098/rspb.2003.2480 PMC1691491 14561279

[eva12490-bib-0056] Renaut, S. , & Rieseberg, L. H. (2015). The accumulation of deleterious mutations as a consequence of domestication and improvement in sunflowers and other compositae crops. Molecular Biology and Evolution, 32, 2273–2283. 25939650 10.1093/molbev/msv106

[eva12490-bib-0057] Rice, P. , Longden, I. , & Bleasby, A. (2000). EMBOSS: The European molecular biology open software suite. Trends in Genetics, 16, 276–277. 10827456 10.1016/s0168-9525(00)02024-2

[eva12490-bib-0058] Ross‐Ibarra, J. , Morrell, P. L. , & Gaut, B. S. (2007). Plant domestication, a unique opportunity to identify the genetic basis of adaptation. Proceedings of the National Academy of Sciences, 104(Suppl 1), 8641–8648. 10.1073/pnas.0700643104 PMC1876441 17494757

[eva12490-bib-0059] Seddon, P. J. , Armstrong, D. P. , & Maloney, R. F. (2007). Developing the science of reintroduction biology. Conservation Biology, 21, 303–312. 17391180 10.1111/j.1523-1739.2006.00627.x

[eva12490-bib-0060] Shafer, A. B. A. , Wolf, J. B. W. , Alves, P. C. , Bergström, L. , Bruford, M. W. , Brännström, I. , … Zieliński, P. (2015). Genomics and the challenging translation into conservation practice. Trends in Ecology & Evolution, 30, 78–87. 25534246 10.1016/j.tree.2014.11.009

[eva12490-bib-0061] Stamatakis, A. (2006). RAxML‐VI‐HPC: Maximum likelihood‐based phylogenetic analyses with thousands of taxa and mixed models. Bioinformatics, 22, 2688–2690. 16928733 10.1093/bioinformatics/btl446

[eva12490-bib-0062] Stamatakis, A. , Hoover, P. , & Rougemont, J. (2008). A rapid bootstrap algorithm for the RAxML web servers. Systematic Biology, 57, 758–771. 18853362 10.1080/10635150802429642

[eva12490-bib-0063] Strzała, T. , Kowalczyk, A. , & Łukaszewicz, E. (2015). Reintroduction of the European capercaillie from the capercaillie breeding centre in Wisła Forest district: Genetic assessments of captive and reintroduced populations. PLoS One, 10, e0145433. 26682897 10.1371/journal.pone.0145433 PMC4684292

[eva12490-bib-0064] Stuwe, M. , & Nievergelt, B. (1991). Recovery of Alpine Ibex from near extinction—The result of effective protection, captive breeding, and reintroductions. Applied Animal Behaviour Science, 29, 379–387.

[eva12490-bib-0065] Tracy, L. N. , Wallis, G. P. , Efford, M. G. , & Jamieson, I. G. (2011). Preserving genetic diversity in threatened species reintroductions: How many individuals should be released? Animal Conservation, 14, 439–446.

[eva12490-bib-0066] Walling, C. A. , Nussey, D. H. , Morris, A. , Clutton‐Brock, T. H. , Kruuk, L. E. B. , & Pemberton, J. M. (2011). Inbreeding depression in red deer calves. Bmc Evolutionary Biology, 11, https://doi.org/10.1186/1471-2148-11-318 10.1186/1471-2148-11-318 PMC3226574 22039837

[eva12490-bib-0067] Waples, R. S. , & Do, C. (2008). ldne: A program for estimating effective population size from data on linkage disequilibrium. Molecular Ecology Resources, 8, 753–756. 21585883 10.1111/j.1755-0998.2007.02061.x

[eva12490-bib-0068] Wright, S. (1922). Coefficients of inbreeding and relationship. The American Naturalist, 56, 330–338.

[eva12490-bib-0069] Wright, S. (1969). The theory of gene frequencies, Vol. 2 Evolution and the Genetics of Populations. Chicago, IL: University of Chicago Press.

[eva12490-bib-0070] Xue, Y. , Prado‐Martinez, J. , Sudmant, P. H. , Narasimhan, V. , Ayub, Q. , Szpak, M. , … Scally, A. (2015). Mountain gorilla genomes reveal the impact of long‐term population decline and inbreeding. Science, 348, 242–245. 25859046 10.1126/science.aaa3952 PMC4668944

[eva12490-bib-0071] Zeder, M. A. , Emshwiller, E. , Smith, B. D. , & Bradley, D. G. (2006). Documenting domestication: The intersection of genetics and archaeology. Trends in Genetics, 22, 139–155. 16458995 10.1016/j.tig.2006.01.007

[eva12490-bib-0072] Zheng, X. , Levine, D. , Shen, J. , Gogarten, S. M. , Laurie, C. , & Weir, B. S. (2012). A high‐performance computing toolset for relatedness and principal component analysis of SNP data. Bioinformatics, 28, 3326–3328. 23060615 10.1093/bioinformatics/bts606 PMC3519454

