# Supplementary material for: Population genomics analyses of European ibex species show lower diversity and higher inbreeding in reintroduced populations
Source: Evol Appl. 2017 Oct 27;11(2):123–39. doi: 10.1111/eva.12490 (PMC5775499; doi:10.1111/eva.12490)
Supplement: Supplementary file 1 [file EVA-11-123-s001.pdf]

SUPPORTING INFORMATION

Population genomics analyses of European ibex species show lower diversity and higher inbreeding in reintroduced populations

Supplementary Figures

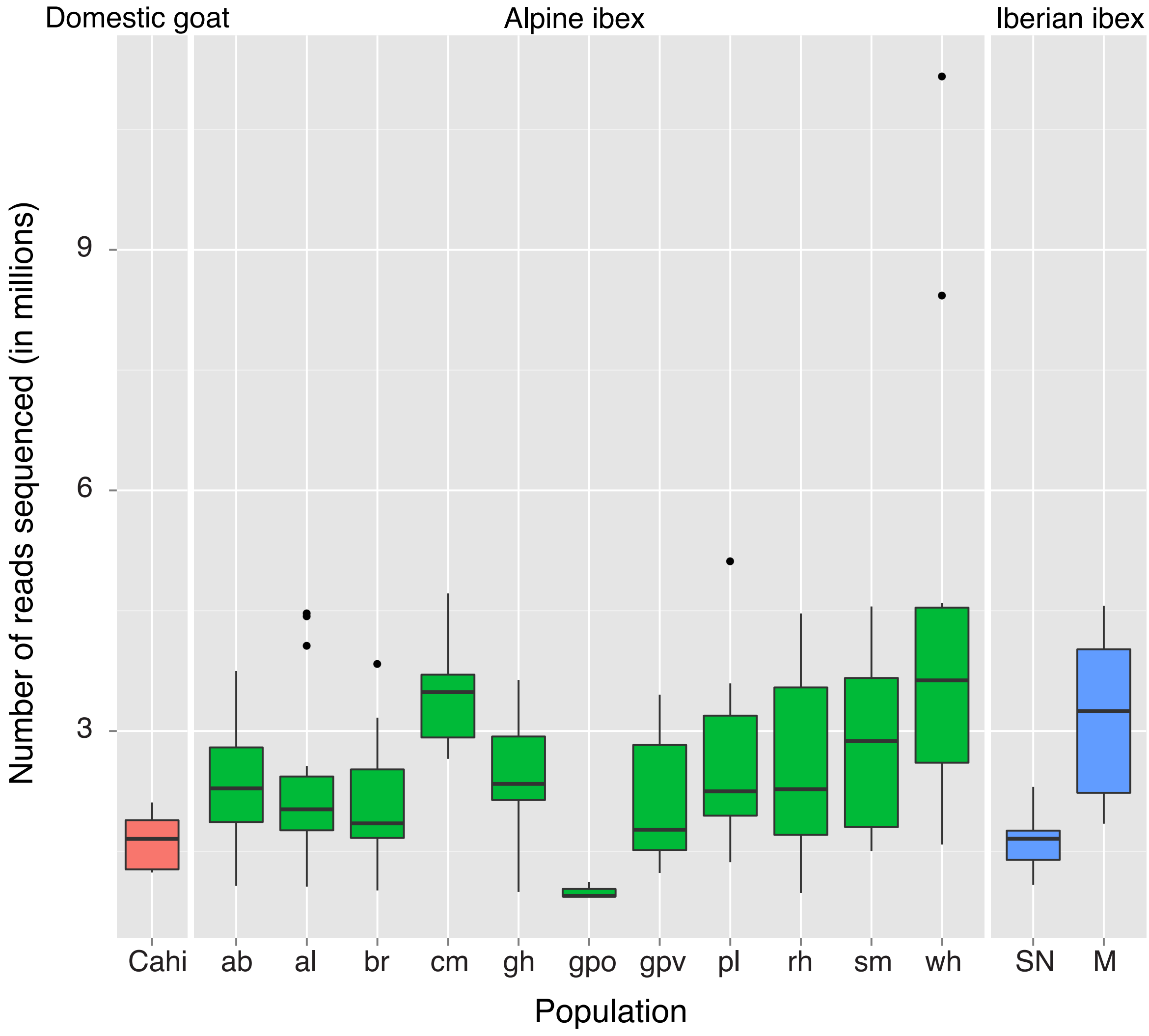

Figure S1: Number of restriction-associated DNA sequencing reads obtained per individual summarized by population. Ibex populations: gp: Gran Paradiso (gpo: sampling location Orco, gpv: sampling location Valsaveranche), al: Albris, br: Brienzer Rothorn, pl: Pleureur, ab: Aletsch-Bietschhorn, sm: Schwarz Mönch, cm: Cape au Moine, gh: Graue Hörner, rh: Rheinwald, wh: Weisshorn, SN: Sierra Nevada, M: Maestrazgo.

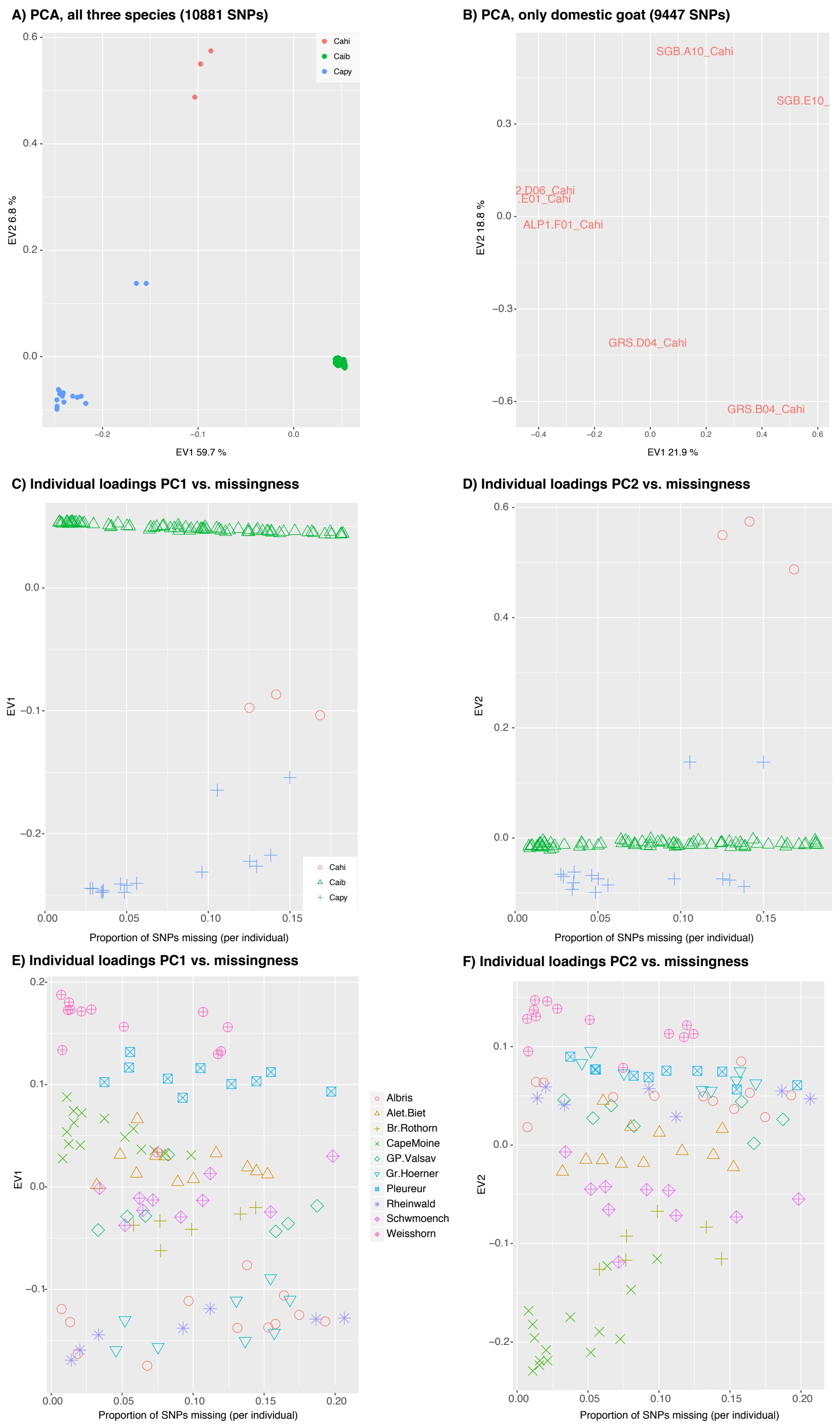

Figure S2: Principal component analyses (PCA).

A) PCA of domestic goat, Iberian ibex and Alpine ibex. B) PCA of domestic goat. C) and D) Correlation of individual loadings on PC1 (C) and PC2 (D) with genotype missingness for domestic goat, Iberian ibex and Alpine ibex. The genotype missingness was calculated as the number of missing genotypes divided by the number of loci included in the analysis. E) and F) Correlation of individual loadings on PC1 (E) and PC2 (F), and genotype missingness for Alpine ibex only.

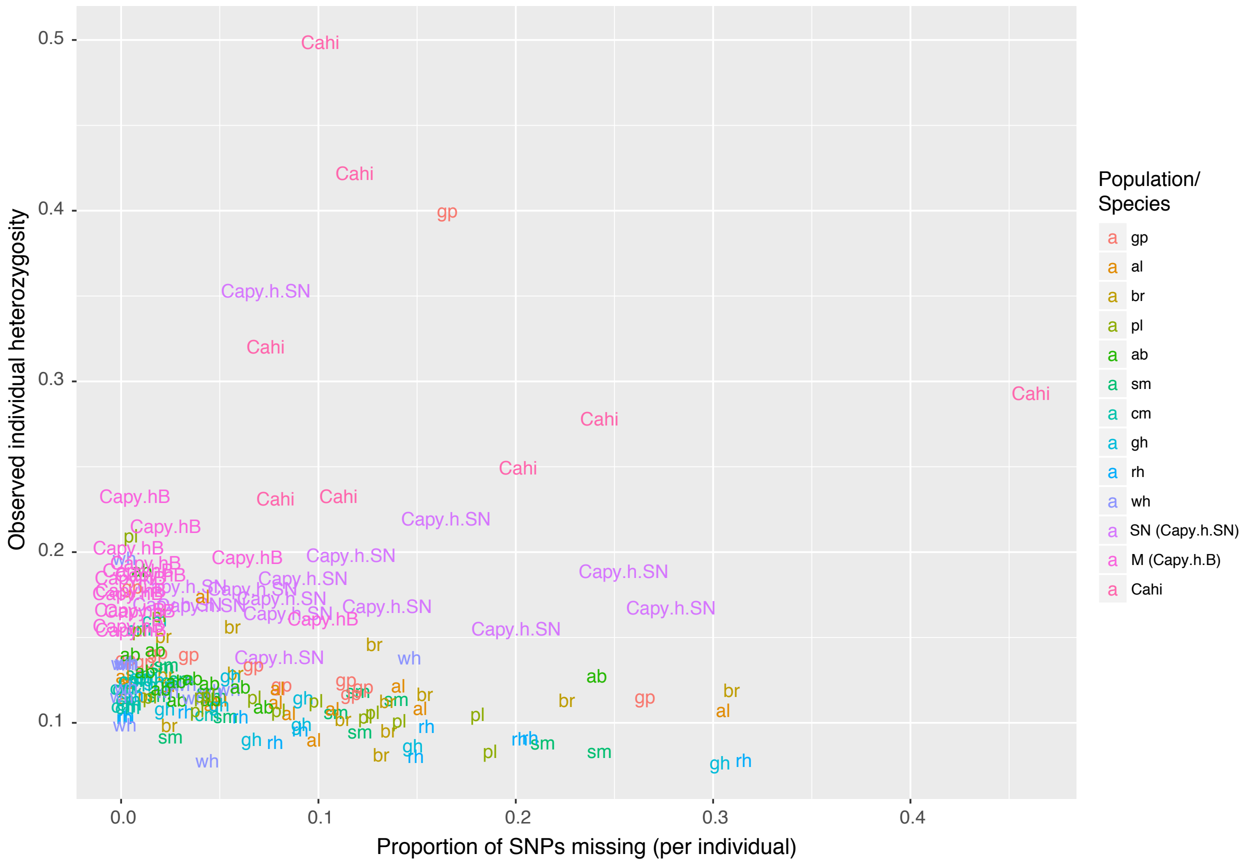

Figure S3: Relationship of observed individual multilocus heterozygosity and genotype missingness. The genotype missingness was calculated as the number of missing genotypes divided by the number of loci included in the analysis. Population abbreviations as in Figure S1.

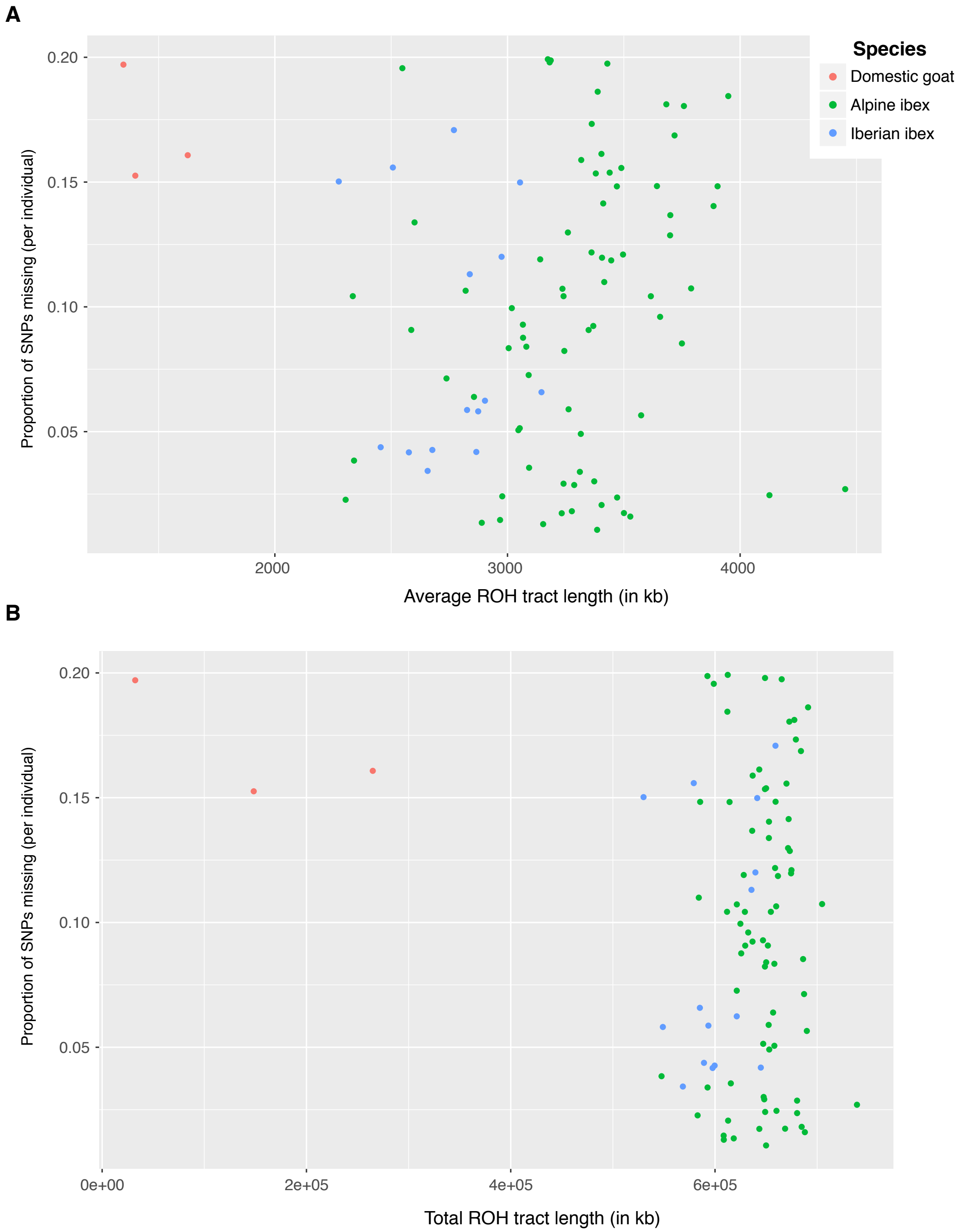

Figure S4: Relationship of genotype missingness and runs of homozygosity. The genotype missingness was calculated as the number of missing genotypes divided by the number of loci included in the analysis. A) Correlation with the average ROH tract length in kilobases per individual. B) Correlation with total ROH tract length in kilobases per individual. ROH of all length categories were included.

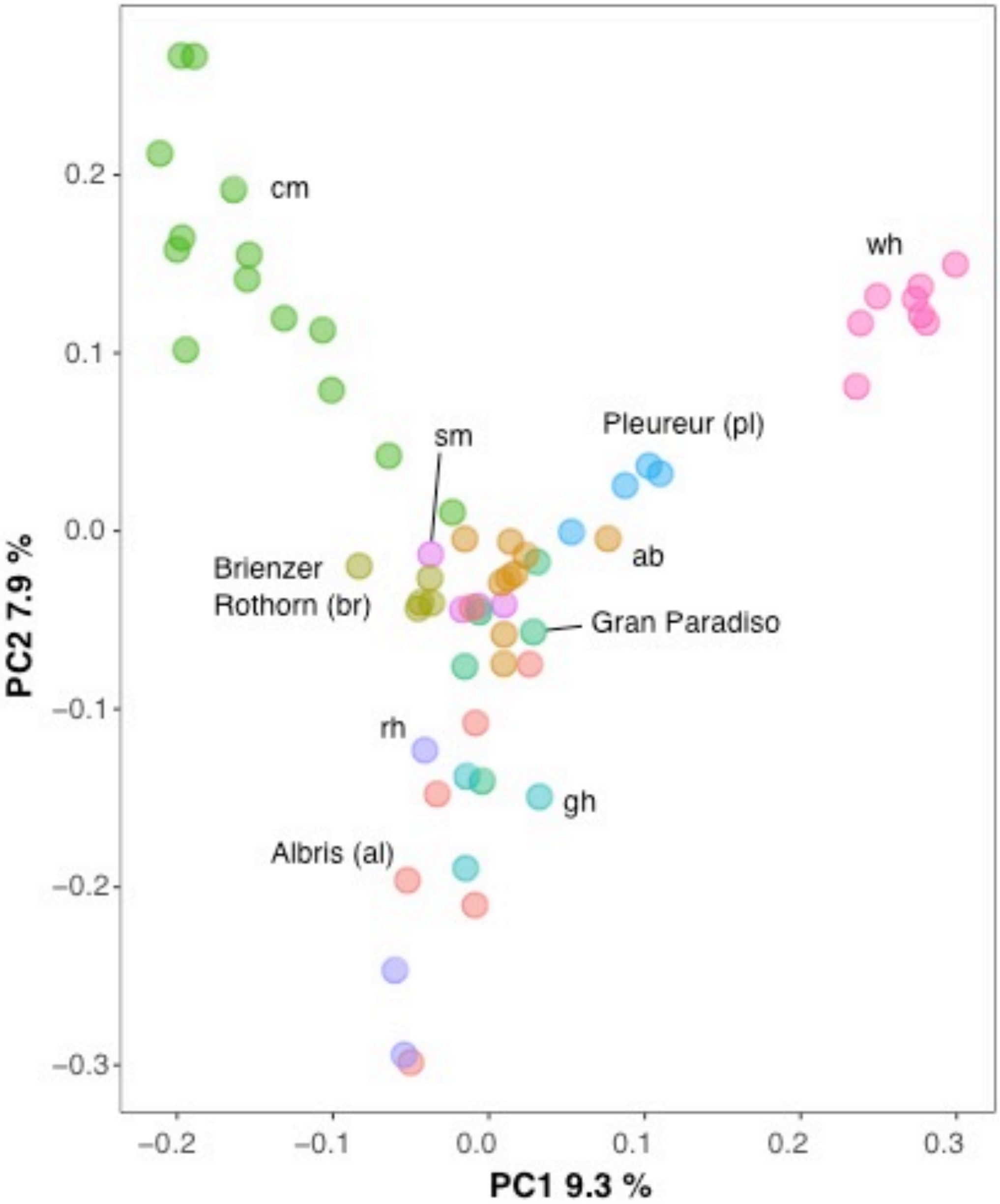

Figure S5: Principal component analysis (PCA) of Alpine ibex. Compared to Figure 2, the PCA was built from single nucleotide polymorphism (SNP) data generated from sequencing reads, which were filtered for PCR duplicates. Please note that the principal axes are rotated compared to Figure 2. The PCA confirmed the presence of three genetic groups of Alpine ibex (represented by three "arms"). Cape au Moine and Weisshorn each occupy a different tip of an arm. The corresponding source populations Brienzer Rothorn and Pleureur are closer to the center. Albris is grouped with secondary populations (Rheinwaldhorn and Graue Hörner) introduced from Albris itself on the third arm. Population abbreviations as in Figure S1.

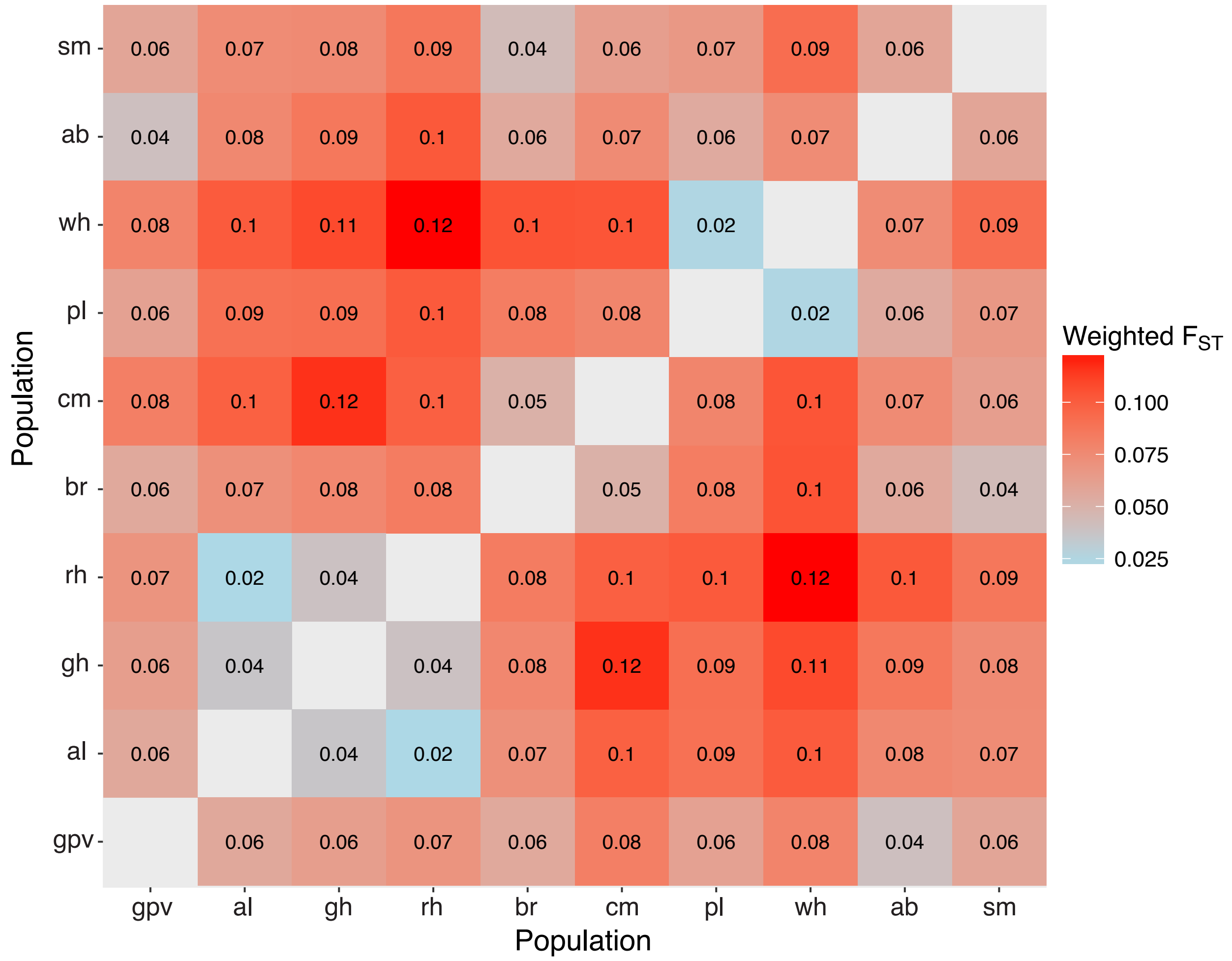

Figure S6: Pairwise F_{ST} among all Alpine ibex populations. Colors indicate different levels of population differentiation. Population abbreviations as in Figure S1.

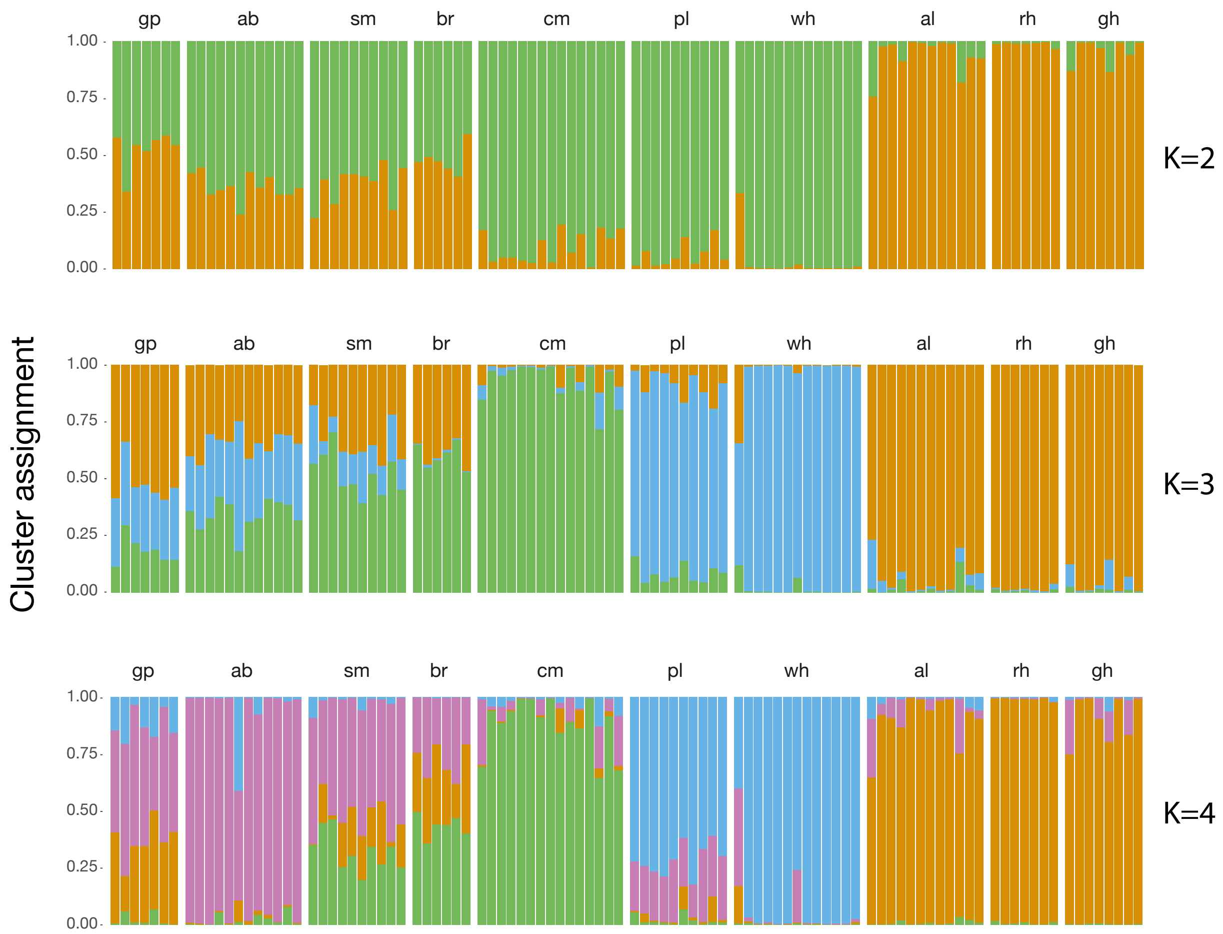

Figure S7: Bayesian clustering analysis (STRUCTURE) of Alpine ibex shown for K=2, 3 and 4 clusters. The color bars show for each individual the posterior probability assignments to the different clusters. Population abbreviations as in Figure S1.

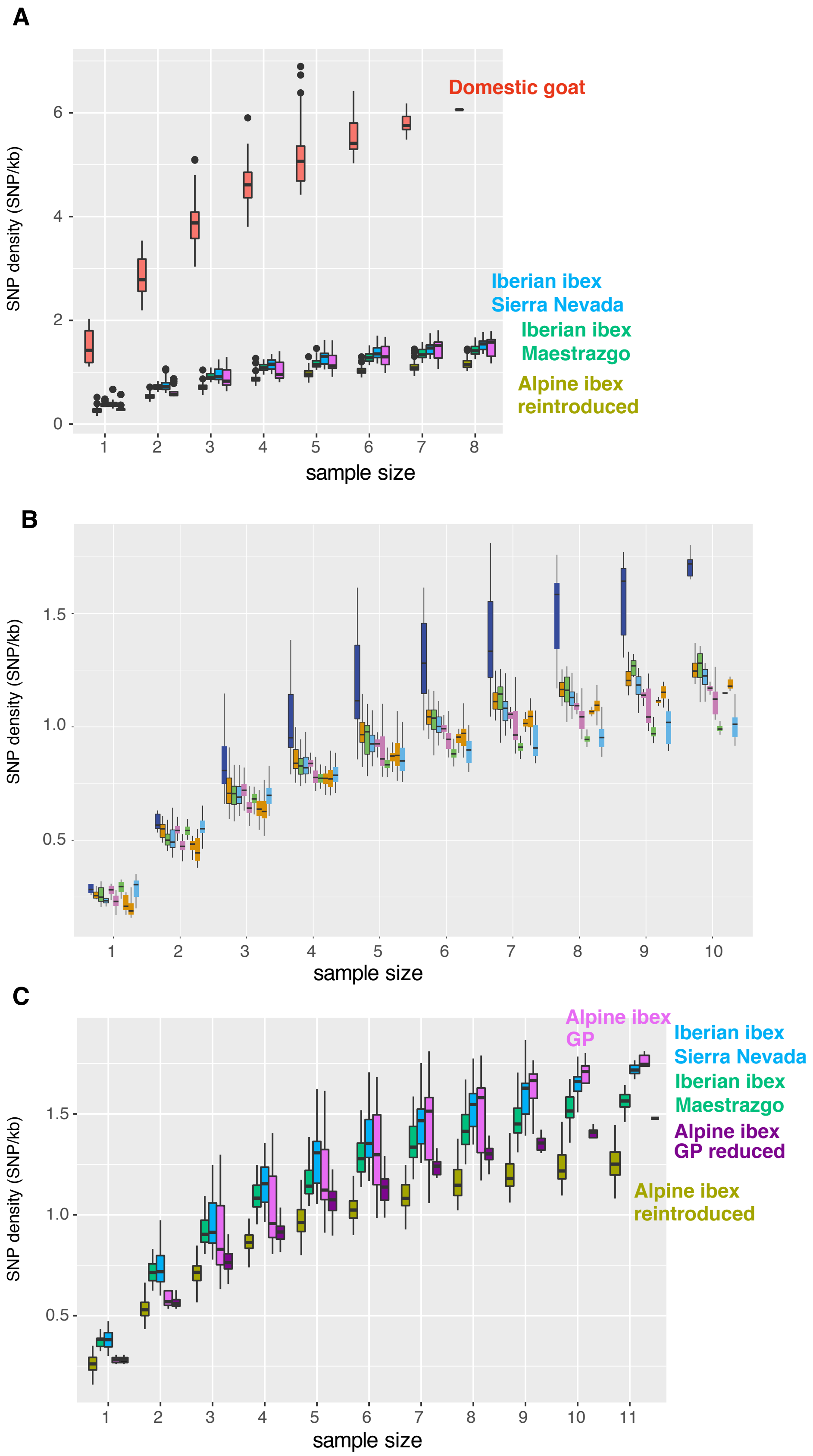
A
Domestic goat
Iberian ibex
Sierra Nevada
Iberian ibex
Maestrazgo
Alpine ibex
reintroduced
SNP density (SNP/kb)
sample size
B
SNP density (SNP/kb)
sample size
C
Alpine ibex
GP
Iberian ibex
Sierra Nevada
Iberian ibex
Maestrazgo
Alpine ibex
GP reduced
Alpine ibex
reintroduced
SNP density (SNP/kb)
sample size

Figure S8: Estimates for total genetic diversity using resampling. Boxplots summarize 100 resampled SNP densities (SNP per kilobase of sequence) for sample sizes between 1 and 11 (depending on sample size of smallest subgroup). A) Resampling curves for domestic goat, Alpine ibex and Iberian ibex. B) Resampling curves per each population of Alpine ibex. Boxplots for each sample size show the following populations from left to right: Gran Paradiso, Albris, Brienzer-Rothorn, Pleureur, Aletsch-Bietschhorn, Schwarz Mönch, Cape au Moine, Graue Hörner, Rheinwaldhorn, Weisshorn. C) Resampling curves of Alpine ibex and Iberian ibex. One Gran Paradiso individual with high heterozygosity was excluded.

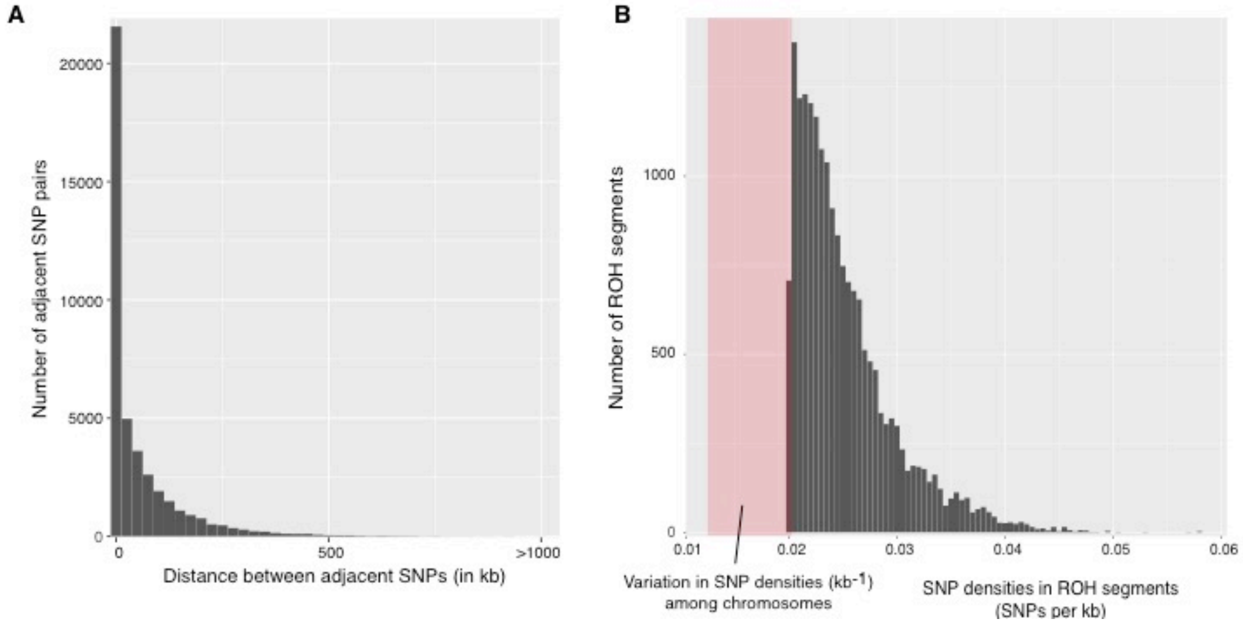

Figure S9: A) Distribution of distance between adjacent SNPs (in kb) along the chromosomes. B) Histogram of SNP densities (SNPs per kb) in runs of homozygosity (ROH) segments. The red area indicates the spread of average SNP densities per chromosome. Note that ROH segments were called predominantly in chromosomal regions with above-average SNP densities.

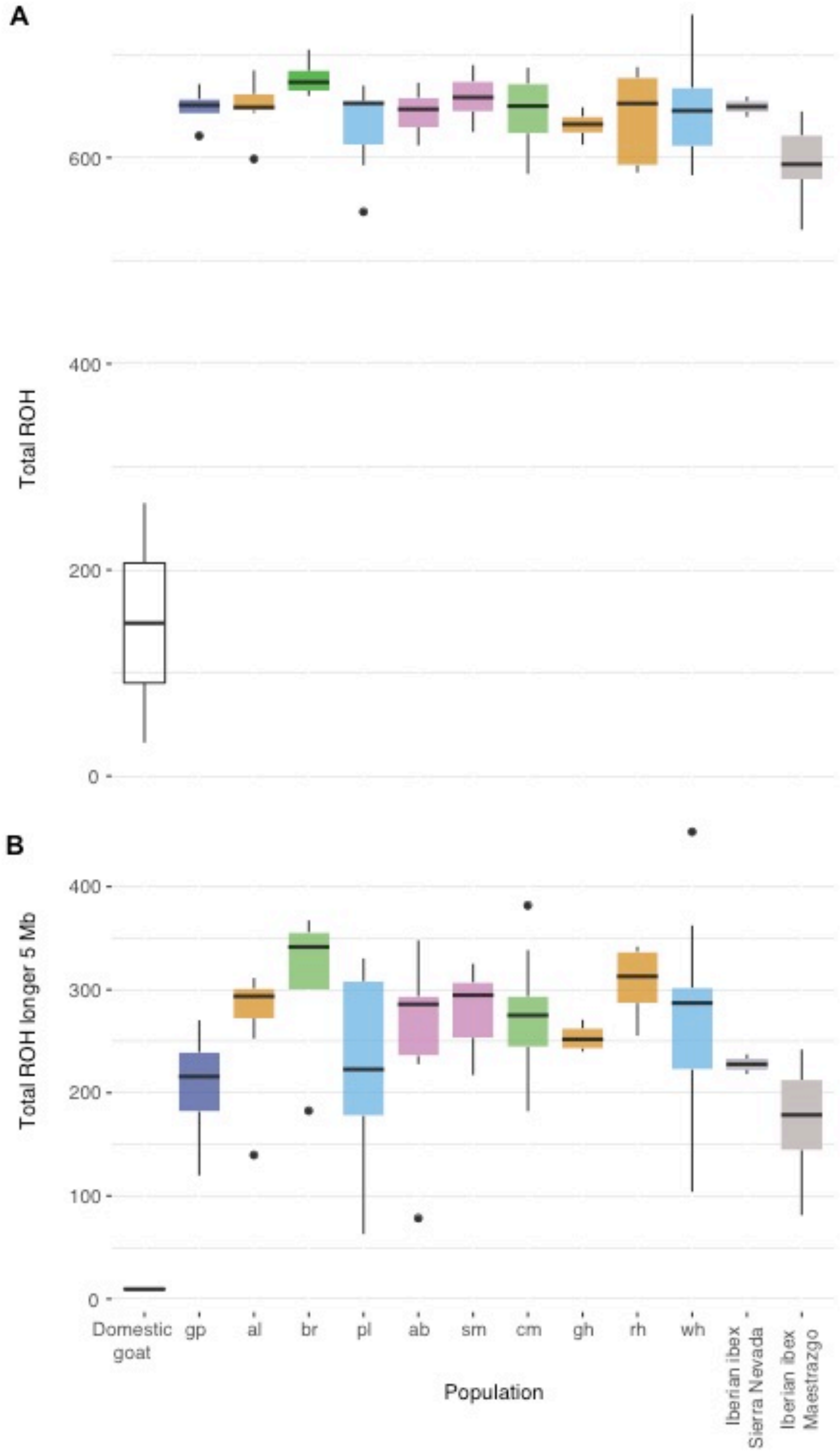

Figure S10: Total length of runs of homozygosity (ROH) per individual summarized by population including (A) ROH of all lengths categories and (B) only ROH longer than 5 Mb. Population abbreviations as in Figure S1.

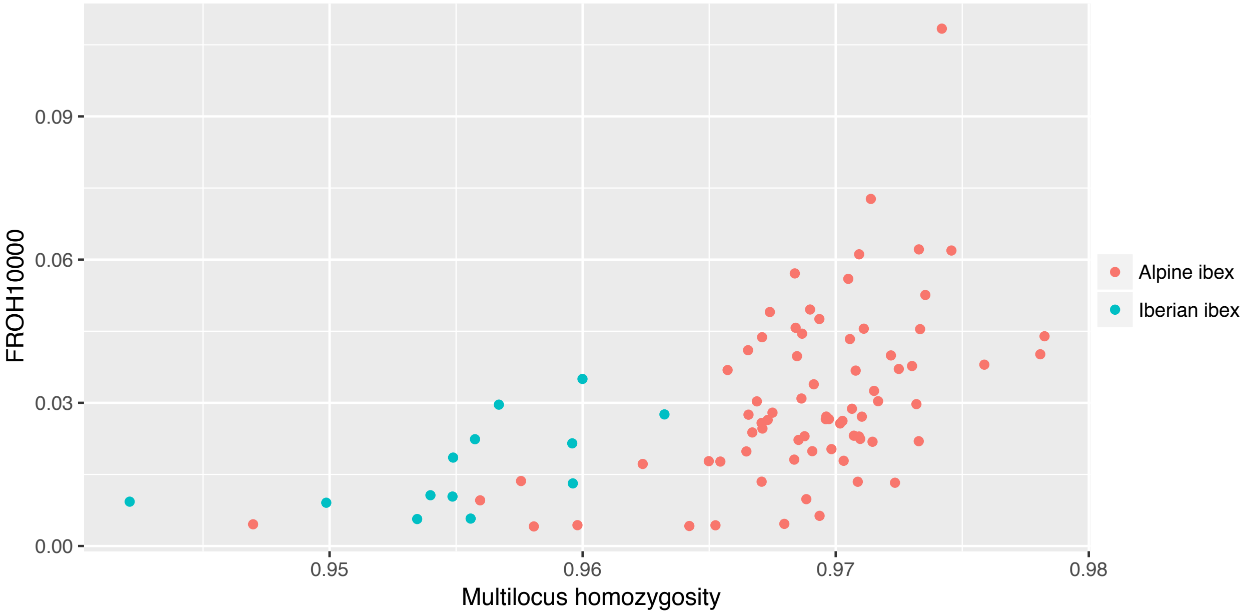

Figure S11: Correlation of multilocus homozygosity and the total length of runs of homozygosity (ROH) per individual. Only ROH longer than 5 Mb were included. Correlations including domestic goat (A) and excluding domestic goat (B) are shown.

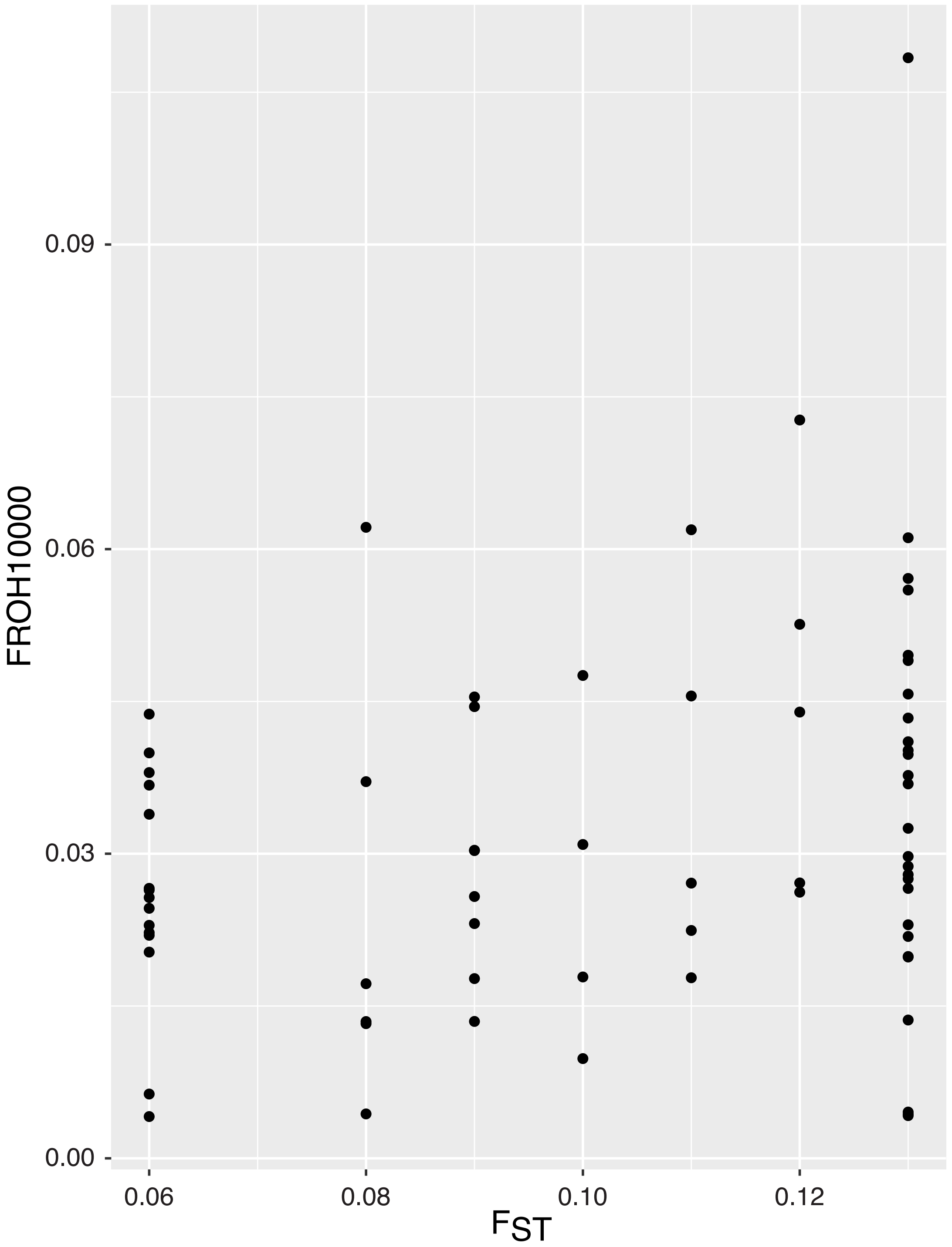

Figure S12: Correlation of the population inbreeding estimated by F_{ST} (Biebach et al. 2010) and ROH-based inbreeding estimates. $FROH_{10000}$ is defined as the total ROH length of ROH longer than 10 Mb divided by the total autosomal genome size.

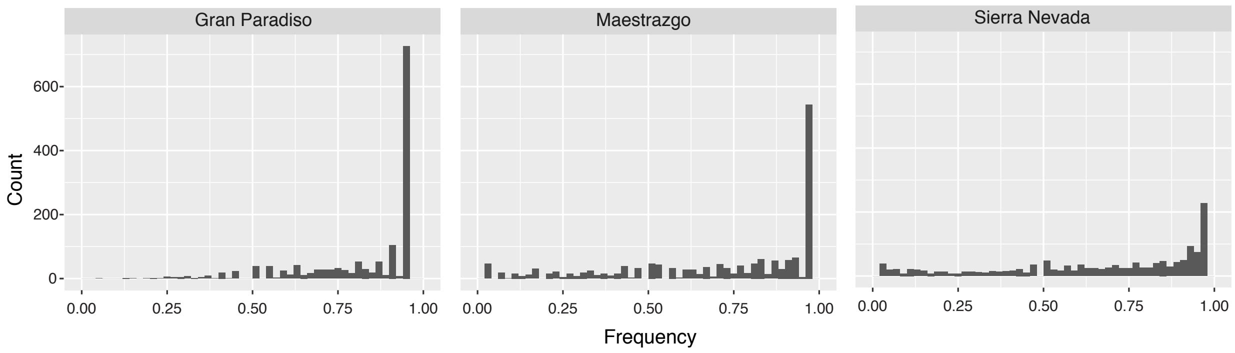

Figure S13: Allele frequency distributions in the Alpine ibex population of Gran Paradiso, and Iberian ibex populations of Maestrazgo and Sierra Nevada.

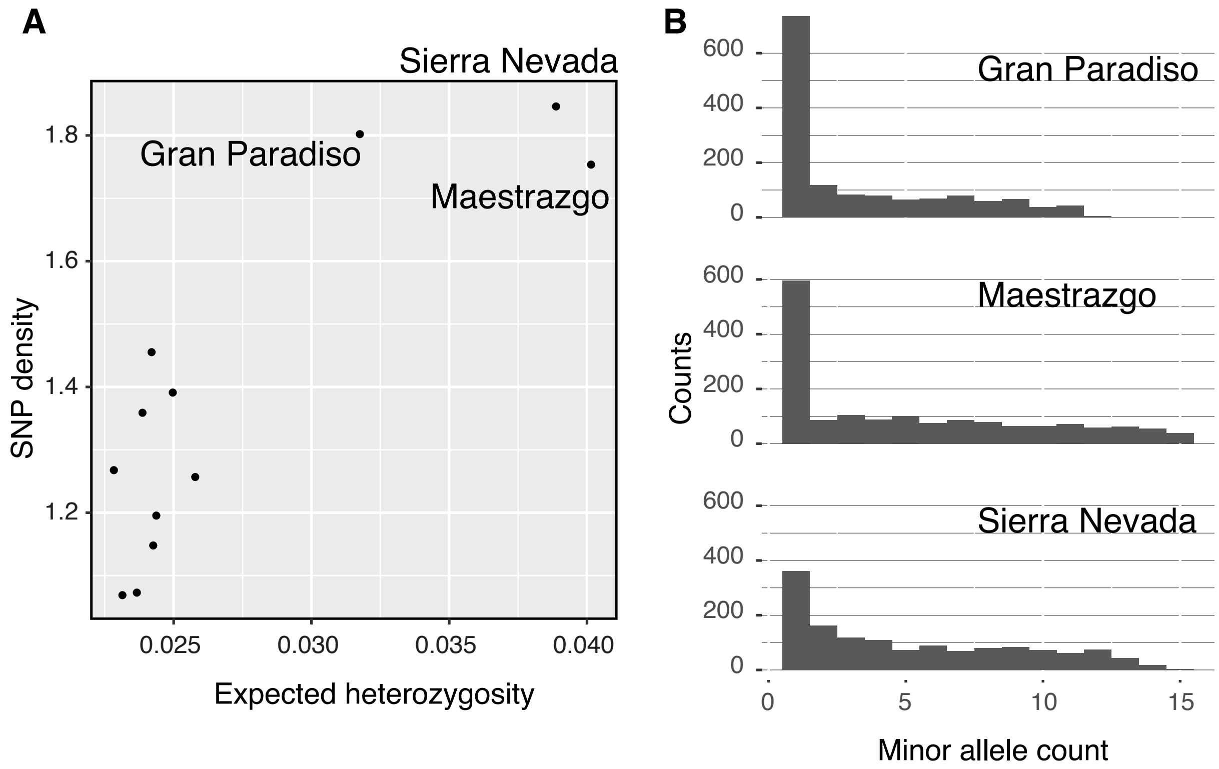

Figure S14: Genetic diversity comparisons.
A) Correlation of the expected heterozygosity and SNP density.
B) Minor allele count distribution of the Gran Paradiso Alpine ibex population and the two Iberian ibex populations Maestrazgo and Sierra Nevada.

Supplementary Tables

Table S1: Data on the reintroduction history of Alpine ibex populations including release date and sex of individuals as well as population coordinates. The coordinates for each population identify the major peak used to label each population. Each population typically occupies a connected area surrounding the mountain.
*Additional individuals were recently introduced into the Pleureur population (pl)

Population	Release period	Population of origin	Males	Females	Sex unknown	Population coordinates	
gp						45°30'52'' N	7°16'11'' E
ab	1932-1979	Zoo Interlaken-Harder, Zoo Peter&Paul, Brienzer Rothorn (br), Mont Pleureur (pl), Arolla	28	29	0	46°23'30'' N	7°51'03'' E
al	1920-1928	Zoo Interlaken-Harder, Zoo Peter&Paul, Swiss Nationalpark	9	9	0	46°27'50'' N	9°57'50'' E
br	1921-1980	Zoo Interlaken-Harder, Zoo Peter&Paul	8	10	0	46°47'14'' N	8°02'49'' E
cm	NA	Wittenberg (founded using animals from Brienzer Rothorn (br) and Mont Pleureur (pl))	NA	NA	NA	46°28'13'' N	6°58'43'' E
gh	1911-1961	Zoo Interlaken-Harder, Zoo Peter&Paul, Albris (al)	25	30	0	46°57'32'' N	9°29'13'' E
pl	1928-1935	Zoo Interlaken-Harder, Zoo Peter&Paul, Gran Paradiso	6	9	6	46°00'58'' N	7°22'09'' E
pl*	2002-2006	Weissmies, Mischabel, Weisshorn, Dents du Midi	27	30	0		
rh	1954	Albris (al)	11	3	0	46°29'38'' N	9°02'25'' E
sm	1924-1950	Zoo Interlaken-Harder, Brienzer Rothorn (br)	14	12	0	46°33'10'' N	7°55'28'' E
wh	1962-1969	Mont Pleureur (pl)	18	6	0	46°06'05'' N	7°42'58'' E

Table S2: Samples included in the study.

SampleName	Population	Species	Scientific name	removed (reason)
GR0001	Albris	Alpine ibex	Capra ibex	
GR0002	Albris	Alpine ibex	Capra ibex	
GR0003	Albris	Alpine ibex	Capra ibex	
GR0004	Albris	Alpine ibex	Capra ibex	
GR0005	Albris	Alpine ibex	Capra ibex	
GR0006	Albris	Alpine ibex	Capra ibex	
GR0007	Albris	Alpine ibex	Capra ibex	
GR0008	Albris	Alpine ibex	Capra ibex	
GR0009	Albris	Alpine ibex	Capra ibex	
GR0010	Albris	Alpine ibex	Capra ibex	
GR0011	Albris	Alpine ibex	Capra ibex	
GR0113	Albris	Alpine ibex	Capra ibex	
GR0201	Albris	Alpine ibex	Capra ibex	
GR0204	Albris	Alpine ibex	Capra ibex	
GR0379	Albris	Alpine ibex	Capra ibex	
VS0013	Aletsch Bietschhorn	Alpine ibex	Capra ibex	
VS0016	Aletsch Bietschhorn	Alpine ibex	Capra ibex	
VS0017	Aletsch Bietschhorn	Alpine ibex	Capra ibex	
VS0043	Aletsch Bietschhorn	Alpine ibex	Capra ibex	
VS0087	Aletsch Bietschhorn	Alpine ibex	Capra ibex	
VS0090	Aletsch Bietschhorn	Alpine ibex	Capra ibex	
VS0435	Aletsch Bietschhorn	Alpine ibex	Capra ibex	missing rate > 0.8
VS0440	Aletsch Bietschhorn	Alpine ibex	Capra ibex	
VS0464	Aletsch Bietschhorn	Alpine ibex	Capra ibex	
VS0465	Aletsch Bietschhorn	Alpine ibex	Capra ibex	
VS0466	Aletsch Bietschhorn	Alpine ibex	Capra ibex	
VS0469	Aletsch Bietschhorn	Alpine ibex	Capra ibex	
VS0471	Aletsch Bietschhorn	Alpine ibex	Capra ibex	
VS0473	Aletsch Bietschhorn	Alpine ibex	Capra ibex	
VS0474	Aletsch Bietschhorn	Alpine ibex	Capra ibex	read count < 900000
BE0002	Brienzer Rothorn	Alpine ibex	Capra ibex	
BE0007	Brienzer Rothorn	Alpine ibex	Capra ibex	
BE0272	Brienzer Rothorn	Alpine ibex	Capra ibex	
BE0273	Brienzer Rothorn	Alpine ibex	Capra ibex	
BE0274	Brienzer Rothorn	Alpine ibex	Capra ibex	
BE0279	Brienzer Rothorn	Alpine ibex	Capra ibex	
BE0280	Brienzer Rothorn	Alpine ibex	Capra ibex	missing rate > 0.8
BE0281	Brienzer Rothorn	Alpine ibex	Capra ibex	
BE0282	Brienzer Rothorn	Alpine ibex	Capra ibex	
BE0430	Brienzer Rothorn	Alpine ibex	Capra ibex	
BE0433	Brienzer Rothorn	Alpine ibex	Capra ibex	
BE0435	Brienzer Rothorn	Alpine ibex	Capra ibex	
BE0436	Brienzer Rothorn	Alpine ibex	Capra ibex	
OW0003	Brienzer Rothorn	Alpine ibex	Capra ibex	
OW0004	Brienzer Rothorn	Alpine ibex	Capra ibex	
VD0007	Cape au Moine	Alpine ibex	Capra ibex	
VD0023	Cape au Moine	Alpine ibex	Capra ibex	
VD0030	Cape au Moine	Alpine ibex	Capra ibex	
VD0038	Cape au Moine	Alpine ibex	Capra ibex	
VD0039	Cape au Moine	Alpine ibex	Capra ibex	
VD0048	Cape au Moine	Alpine ibex	Capra ibex	
VD0052	Cape au Moine	Alpine ibex	Capra ibex	
VD0057	Cape au Moine	Alpine ibex	Capra ibex	
VD0058	Cape au Moine	Alpine ibex	Capra ibex	
VD0059	Cape au Moine	Alpine ibex	Capra ibex	
VD0060	Cape au Moine	Alpine ibex	Capra ibex	
VD0067	Cape au Moine	Alpine ibex	Capra ibex	
VD0142	Cape au Moine	Alpine ibex	Capra ibex	
VD0147	Cape au Moine	Alpine ibex	Capra ibex	
VD0208	Cape au Moine	Alpine ibex	Capra ibex	
**GPO03D**	**Gran Paradiso Orco**	**Alpine ibex**	**Capra ibex**	**very high heterozygosity**
GPO09D_1	Gran Paradiso Orco	Alpine ibex	Capra ibex	missing rate > 0.8
GPO39B	Gran Paradiso Orco	Alpine ibex	Capra ibex	missing rate > 0.8
GPV02C	Gran Paradiso Valsav	Alpine ibex	Capra ibex	read count < 900000
GPV07D_1	Gran Paradiso Valsav	Alpine ibex	Capra ibex	
GPV07D_2	Gran Paradiso Valsav	Alpine ibex	Capra ibex	
GPV08C	Gran Paradiso Valsav	Alpine ibex	Capra ibex	
GPV099	Gran Paradiso Valsav	Alpine ibex	Capra ibex	
GPV10C	Gran Paradiso Valsav	Alpine ibex	Capra ibex	
GPV12C	Gran Paradiso Valsav	Alpine ibex	Capra ibex	
GPV13C	Gran Paradiso Valsav	Alpine ibex	Capra ibex	
GPV14C	Gran Paradiso Valsav	Alpine ibex	Capra ibex	
GPV14D	Gran Paradiso Valsav	Alpine ibex	Capra ibex	
GPV15C	Gran Paradiso Valsav	Alpine ibex	Capra ibex	
GPV16C	Gran Paradiso Valsav	Alpine ibex	Capra ibex	
GPV19D	Gran Paradiso Valsav	Alpine ibex	Capra ibex	
SG0012	Graue Hörner	Alpine ibex	Capra ibex	
SG0013	Graue Hörner	Alpine ibex	Capra ibex	
SG0015	Graue Hörner	Alpine ibex	Capra ibex	
SG0016	Graue Hörner	Alpine ibex	Capra ibex	
SG0025	Graue Hörner	Alpine ibex	Capra ibex	
SG0032	Graue Hörner	Alpine ibex	Capra ibex	
SG0033	Graue Hörner	Alpine ibex	Capra ibex	
SG0046	Graue Hörner	Alpine ibex	Capra ibex	
SG0205	Graue Hörner	Alpine ibex	Capra ibex	read count < 900000
SG0206	Graue Hörner	Alpine ibex	Capra ibex	read count < 900000
SG0214	Graue Hörner	Alpine ibex	Capra ibex	read count < 900000
SG0216	Graue Hörner	Alpine ibex	Capra ibex	
SG0217	Graue Hörner	Alpine ibex	Capra ibex	read count < 900000
SG0219	Graue Hörner	Alpine ibex	Capra ibex	
SG0221	Graue Hörner	Alpine ibex	Capra ibex	
VS0140	Pleureur	Alpine ibex	Capra ibex	
VS0141	Pleureur	Alpine ibex	Capra ibex	
VS0280	Pleureur	Alpine ibex	Capra ibex	
VS0308	Pleureur	Alpine ibex	Capra ibex	
VS0313	Pleureur	Alpine ibex	Capra ibex	
VS0329	Pleureur	Alpine ibex	Capra ibex	
VS0330	Pleureur	Alpine ibex	Capra ibex	
VS0856	Pleureur	Alpine ibex	Capra ibex	
VS0882	Pleureur	Alpine ibex	Capra ibex	
VS0983	Pleureur	Alpine ibex	Capra ibex	
VS1120	Pleureur	Alpine ibex	Capra ibex	
VS1121	Pleureur	Alpine ibex	Capra ibex	
VS1123	Pleureur	Alpine ibex	Capra ibex	
VS1171	Pleureur	Alpine ibex	Capra ibex	
VS1212	Pleureur	Alpine ibex	Capra ibex	
GR0140	Rheinwald	Alpine ibex	Capra ibex	read count < 900000
GR0310	Rheinwald	Alpine ibex	Capra ibex	
GR0721	Rheinwald	Alpine ibex	Capra ibex	
GR0727	Rheinwald	Alpine ibex	Capra ibex	read count < 900000
GR0728	Rheinwald	Alpine ibex	Capra ibex	
GR0729	Rheinwald	Alpine ibex	Capra ibex	read count < 900000
GR0732	Rheinwald	Alpine ibex	Capra ibex	
GR0766	Rheinwald	Alpine ibex	Capra ibex	
GR1380	Rheinwald	Alpine ibex	Capra ibex	
GR1387	Rheinwald	Alpine ibex	Capra ibex	
GR1390	Rheinwald	Alpine ibex	Capra ibex	
GR1422	Rheinwald	Alpine ibex	Capra ibex	
GR1424	Rheinwald	Alpine ibex	Capra ibex	
GR1709	Rheinwald	Alpine ibex	Capra ibex	
GR1728	Rheinwald	Alpine ibex	Capra ibex	
BE0125	Schwarz Moench	Alpine ibex	Capra ibex	
BE0129	Schwarz Moench	Alpine ibex	Capra ibex	
BE0143	Schwarz Moench	Alpine ibex	Capra ibex	
BE0146	Schwarz Moench	Alpine ibex	Capra ibex	
BE0148	Schwarz Moench	Alpine ibex	Capra ibex	
BE0320	Schwarz Moench	Alpine ibex	Capra ibex	
BE0321	Schwarz Moench	Alpine ibex	Capra ibex	
BE0322	Schwarz Moench	Alpine ibex	Capra ibex	
BE0323	Schwarz Moench	Alpine ibex	Capra ibex	
BE0324	Schwarz Moench	Alpine ibex	Capra ibex	
BE0327	Schwarz Moench	Alpine ibex	Capra ibex	
BE0328	Schwarz Moench	Alpine ibex	Capra ibex	
BE0331	Schwarz Moench	Alpine ibex	Capra ibex	
BE0333	Schwarz Moench	Alpine ibex	Capra ibex	
BE0336	Schwarz Moench	Alpine ibex	Capra ibex	
VS0003	Weisshorn	Alpine ibex	Capra ibex	
VS0031	Weisshorn	Alpine ibex	Capra ibex	
VS0034	Weisshorn	Alpine ibex	Capra ibex	
VS0035	Weisshorn	Alpine ibex	Capra ibex	
VS0037	Weisshorn	Alpine ibex	Capra ibex	
VS0064	Weisshorn	Alpine ibex	Capra ibex	
VS0079	Weisshorn	Alpine ibex	Capra ibex	
VS0081	Weisshorn	Alpine ibex	Capra ibex	
VS0082	Weisshorn	Alpine ibex	Capra ibex	
VS0295	Weisshorn	Alpine ibex	Capra ibex	
VS0491	Weisshorn	Alpine ibex	Capra ibex	
VS0492	Weisshorn	Alpine ibex	Capra ibex	
VS0497	Weisshorn	Alpine ibex	Capra ibex	read count < 900000
VS0515	Weisshorn	Alpine ibex	Capra ibex	
VS0627	Weisshorn	Alpine ibex	Capra ibex	
ALP1_E01	Capra hircus	Domestic goat	Capra hircus	
ALP1_F01	Capra hircus	Domestic goat	Capra hircus	
ALP2_D06	Capra hircus	Domestic goat	Capra hircus	
GRS_B04	Capra hircus	Domestic goat	Capra hircus	
GRS_D04	Capra hircus	Domestic goat	Capra hircus	
PCG_D06	Capra hircus	Domestic goat	Capra hircus	
PCG_E06	Capra hircus	Domestic goat	Capra hircus	read count < 900000
SGB_A10	Capra hircus	Domestic goat	Capra hircus	
SGB_E10	Capra hircus	Domestic goat	Capra hircus	
Z17	Barcelona	Spanish ibex	Capra pyrenaica hispanica	
Z19	Barcelona	Spanish ibex	Capra pyrenaica hispanica	
Z20	Barcelona	Spanish ibex	Capra pyrenaica hispanica	
Z21	Barcelona	Spanish ibex	Capra pyrenaica hispanica	
Z23	Barcelona	Spanish ibex	Capra pyrenaica hispanica	
Z30	Barcelona	Spanish ibex	Capra pyrenaica hispanica	
Z31	Barcelona	Spanish ibex	Capra pyrenaica hispanica	
Z33	Barcelona	Spanish ibex	Capra pyrenaica hispanica	
Z34	Barcelona	Spanish ibex	Capra pyrenaica hispanica	
Z35	Barcelona	Spanish ibex	Capra pyrenaica hispanica	
Z37	Barcelona	Spanish ibex	Capra pyrenaica hispanica	
Z39	Barcelona	Spanish ibex	Capra pyrenaica hispanica	
Z5	Barcelona	Spanish ibex	Capra pyrenaica hispanica	
Z6	Barcelona	Spanish ibex	Capra pyrenaica hispanica	
Z8	Barcelona	Spanish ibex	Capra pyrenaica hispanica	
AL520	Sierra Nevada	Spanish ibex	Capra pyrenaica hispanica	
AL528	Sierra Nevada	Spanish ibex	Capra pyrenaica hispanica	
AL529	Sierra Nevada	Spanish ibex	Capra pyrenaica hispanica	
AL554	Sierra Nevada	Spanish ibex	Capra pyrenaica hispanica	
AL562	Sierra Nevada	Spanish ibex	Capra pyrenaica hispanica	
AL565	Sierra Nevada	Spanish ibex	Capra pyrenaica hispanica	
AL596	Sierra Nevada	Spanish ibex	Capra pyrenaica hispanica	
AL599	Sierra Nevada	Spanish ibex	Capra pyrenaica hispanica	
AL600	Sierra Nevada	Spanish ibex	Capra pyrenaica hispanica	
ALP199	Sierra Nevada	Spanish ibex	Capra pyrenaica hispanica	
ALP222	Sierra Nevada	Spanish ibex	Capra pyrenaica hispanica	
M139	Sierra Nevada	Spanish ibex	Capra pyrenaica hispanica	
M238	Sierra Nevada	Spanish ibex	Capra pyrenaica hispanica	
M518	Sierra Nevada	Spanish ibex	Capra pyrenaica hispanica	
P252	Sierra Nevada	Spanish ibex	Capra pyrenaica hispanica	

Table S3: Analyses of molecular variance (AMOVA) among Alpine ibex populations. With the exception of two, all secondary reintroduced populations in this study were established from one primary reintroduced population. For the lower hierarchical level, we grouped each secondary reintroduced population with its primary population if there was such a simple one-to-one relationship. For the higher hierarchical level, we grouped all 1-to-1 pairs of reintroduced populations mentioned above and compared these with the source population (Gran Paradiso).

**Source of variation**	**Degrees of freedom**	**Sum of squares**	**Percentage of variation**	***p* value**
Between source-reintroduced pop.	1	1207.5	-4.5	0.255
Between primary-secondary pop.	2	5978.2	10.0	0.0097
Between populations	4	4272.9	5.1	0.0001
Within populations	70	48792.6	89.4	0.0001
Total	77	60251.3	100.0	-

Table S4: Effective population size (N_e) estimates based on LD with their confidence intervals. N_e estimates were obtained using the software NeEstimator and different subsets of the data with different filters for the minor allele frequency.

Population	Species	maf=0.1	maf=0.05	maf=0.02
gp	Alpine ibex	**1174.8** (169.9, Inf)	**696.2** (173.8, Inf)	**3.1** (3, 3.2)
al	Alpine ibex	**452.8** (171.7, Inf)	**633.2** (205.1, Inf)	**Inf** (519.5, Inf)
pl	Alpine ibex	**254.6** (122.6, Inf)	**543.8** (181.6, Inf)	**Inf** (1097.1, Inf)
br	Alpine ibex	**210.8** (103, Inf)	**322.7** (133.7, Inf)	**1714.6** (243.6, Inf)
sm	Alpine ibex	**121.6** (79.5, 249.4)	**130.1** (87.1, 250.2)	**412.7** (188.6, Inf)
ab	Alpine ibex	**70.8** (55.3, 97.1)	**88.3** (67.7, 125.5)	**128.8** (95.1, 197.6)
cm	Alpine ibex	**82.2** (68.7, 101.9)	**88.4** (74.2, 109)	**131.8** (106.8, 171.3)
rh	Alpine ibex	**88.7** (55.8, 203.1)	**120.2** (71.2, 355)	**122.3** (78.6, 265.1)
wh	Alpine ibex	**40.1** (35.4, 46)	**40** (35.6, 45.4)	**28.4** (26.3, 30.8)
gh	Alpine ibex	**68.1** (48.1, 113.9)	**139.4** (84.4, 379.1)	**139.4** (84.4, 379.1)
SN	Spanish ibex	**1844** (188.1, Inf)	**421.9** (155.6, Inf)	**54.6** (46.3, 66.3)
M	Spanish ibex	**73.3** (63.5, 86.4)	**83.6** (72.2, 99)	**54.8** (51.3, 58.6)
